# Mechanisms Underlying Spontaneous Action Potential Generation Induced by Catecholamine in Pulmonary Vein Cardiomyocytes: A Simulation Study

**DOI:** 10.3390/ijms20122913

**Published:** 2019-06-14

**Authors:** Shohei Umehara, Xiaoqiu Tan, Yosuke Okamoto, Kyoichi Ono, Akinori Noma, Akira Amano, Yukiko Himeno

**Affiliations:** 1 Department of Bioinformatics, College of Life Sciences, Ritsumeikan University, Shiga 525-8577, Japan; shoumezoo@gmail.com (S.U.); noma@sk.ritsumei.ac.jp (A.N.); a-amano@fc.ritsumei.ac.jp (A.A.); 2 Institute of Cardiovascular Research, Southwest Medical University, Luzhou 640000, China; tanxiaoqiu@swmu.edu.cn; 3 Department of Cell Physiology, Graduate School of Medicine, Akita University, Akita 010-8543, Japan; okamoto@med.akita-u.ac.jp (Y.O.); onok@med.akita-u.ac.jp (K.O.)

**Keywords:** rat pulmonary vein cardiac myocyte, automaticity, α1- and β1-adrenoceptor, InsP_3_R, IP_3_

## Abstract

Cardiomyocytes and myocardial sleeves dissociated from pulmonary veins (PVs) potentially generate ectopic automaticity in response to noradrenaline (NA), and thereby trigger atrial fibrillation. We developed a mathematical model of rat PV cardiomyocytes (PVC) based on experimental data that incorporates the microscopic framework of the local control theory of Ca^2+^ release from the sarcoplasmic reticulum (SR), which can generate rhythmic Ca^2+^ release (limit cycle revealed by the bifurcation analysis) when total Ca^2+^ within the cell increased. Ca^2+^ overload in SR increased resting Ca^2+^ efflux through the type II inositol 1,4,5-trisphosphate (IP_3_) receptors (InsP_3_R) as well as ryanodine receptors (RyRs), which finally triggered massive Ca^2+^ release through activation of RyRs via local Ca^2+^ accumulation in the vicinity of RyRs. The new PVC model exhibited a resting potential of −68 mV. Under NA effects, repetitive Ca^2+^ release from SR triggered spontaneous action potentials (APs) by evoking transient depolarizations (TDs) through Na^+^/Ca^2+^ exchanger (AP_TD_s). Marked and variable latencies initiating AP_TD_s could be explained by the time courses of the α1- and β1-adrenergic influence on the regulation of intracellular Ca^2+^ content and random occurrences of spontaneous TD activating the first AP_TD_. Positive and negative feedback relations were clarified under AP_TD_ generation.

## 1. Introduction

Several types of atrial fibrillation may be attributed to the ectopic activity of myocardial cells in the sleeves of pulmonary vein cardiomyocytes (PVCs) under augmented sympathetic stimulation [1,2,3,4,5,6]. Supporting this hypothesis, electrophysiological and histochemical experiments of rat PVCs by Okamoto et al. demonstrated generations of spontaneous action potentials under the influence of noradrenaline (NA) in dissociated PVCs [7,8] as observed in the tissue preparations [9].

The arrhythmogenic influencing of the stimulation of the α1-adrenergic receptor (AR) has been extensively studied in atrial cardiac myocytes [10,11]. These studies have indicated the functional significance of the co-localization of type II inositol 1,4,5-trisphosphate (IP_3_) receptors (InsP_3_Rs) with type II ryanodine receptors (RyRs). The Ca^2+^ release through InsP_3_Rs from the sarcoplasmic reticulum (SR) was suggested to directly evoke a larger Ca^2+^ release via RyRs to initiate Ca^2+^ sparks. If multiple Ca^2+^ sparks overlap others, the resultant Ca^2+^ transient can trigger the temporal depolarization (TD) of the resting membrane through the Na^+^/Ca^2+^ exchanger (NCX), thereby triggering action potentials (AP_TD_s). Co-immunostaining of atrial myocytes with antibodies against type II RyRs and type II InsP_3_Rs revealed that they were localized on the SR membrane facing the subsarcolemmal space. This coupling of InsP_3_R with RyR has scarcely been observed in the free running network of SR, where RyRs are expressed at a lower density. Thus, the excitation–contraction (E–C) coupling in atrial myocytes may be activated if InsP_3_R is activated by the α1-AR stimulation. Spontaneous TD might occur at the junction of the SR terminal cisterna with the cell surface membrane in atrial myocytes.

In contrast to atrial myocytes, the E–C coupling in ventricular myocytes mostly occurs at the dyadic junction all over the cell, but the density of the InsP_3_R expression is much lower than that in the atrial myocytes [10]. The density of the distribution of RyRs in ventricular myocytes is much higher than in the atrial myocytes. Therefore, the probability of direct coupling of RyRs with InsP_3_R might be expected to be much less in ventricular myocytes, where the enhancement of TD is barely evoked by the α1-AR stimulation.

The PVCs in the sleeves of pulmonary veins are comparable in size and shape to ventricular myocytes and have a regular sarcomere pattern. The localization of InsP_3_Rs was demonstrated to be parallel to the sarcomere pattern at a high density by Okamoto et al. [7]. They also demonstrated that the noradrenaline (NA) stimulation evoked a train of AP_TD_ discharge and that the resting membrane potential showed sporadic micro-fluctuations even in the absence of adrenergic stimulation.

Although different cell types might be involved in the arrhythmogenic activity in the pulmonary vein in different species [3], we focused on a particular cell type, which has been well characterized in a rat pulmonary vein [7,8]. We examined mechanisms underlying the generation of a train of spontaneous AP_TD_s in response to NA stimulation by developing a mathematical PVC model. Until now, mathematical modeling of PVC has only been completed in humans [12] and rabbits [13], both of which showed automaticity in the absence of NA stimulation. Conversely, neither cardiomyocytes nor tissue preparation in PVs exhibited automaticity in rats [7,9]. To understand the mechanisms underlying the automaticity induced by NA stimulation by involving mobilization of intracellular Ca^2+^ as suggested by Okamoto et al., we used a refined model of the Ca^2+^-induced Ca^2+^ release (CICR) mechanisms, which was developed based on the Hinch model of Ca^2+^ releasing unit CaRU [14,15] and was adjusted to the human ventricular cell (HuVEC) model [16]. The novel CICR model was adopted in our study in combination with the InsP_3_R model. The desensitization of the response to continuous α1-AR stimulation [17] and β1-AR stimulation [18] were defined by empirical equations. The simulation results showed that the arrhythmogenic activity (spontaneous generation of repetitive AP_TD_) of PVCs could be initiated when variable conditions are combined in addition to the close coupling of InsP_3_R and RyR. The pivotal additional factor is the activation of the β1-AR receptor to increase the Ca^2+^ store within SR (Ca^2+^ overload) through enhancement of both L-type Ca^2+^ channel (LCC) and sarcoplasmic/endoplasmic reticulum calcium pump (SERCA). The whole cell simulation of the PVC model suggests that Ca^2+^ extrusion by NCX, the temporal Ca^2+^ accumulation near the Ca^2+^ releasing site within the cytosol, and a Ca^2+^ overload of the SR are also essential in evoking spontaneous AP_TD_s in the PVCs.

## 2. Results

### 2.1. Intracellular Ca^2+^ Dynamics Revealed by Bifurcation Analyses

Experimental studies have established that spontaneous Ca^2+^ release from SR occurs, and the Ca^2+^ transient evokes TD through the activation of NCX if the cardiac myocyte is overloaded with Ca^2+^ [19,20,21,22]. These cytosolic Ca^2+^ oscillations are important because they are accompanied by spontaneous oscillations in current and membrane potential to produce action potentials [23]. To clarify the behavioral characteristics of the cytosolic [Ca^2+^] in our PVC model, we examined steady-state solutions in the minimal model of intracellular Ca^2+^ dynamics. The constituents of this minimal model are described in Section 4. In short, we removed all the current systems on the plasma membrane from the PVC model to focus on the oscillatory behavior of intracellular Ca^2+^. To this minimal model, we applied bifurcation analyses, which have been successfully used to identify equilibrium points and/or the stable limit cycle in the mathematical whole cell or reduced models [24,25,26].

**Figure 1:**
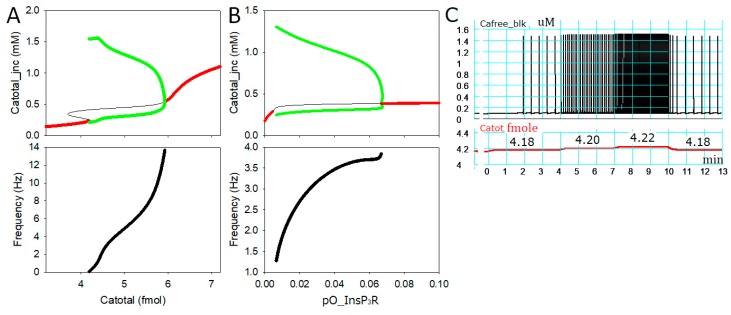
Bifurcation diagrams of the Ca^2+^ dynamics model: (**A**) A bifurcation diagram with the total Ca^2+^ content within the cell on the abscissa and total amount of Ca^2+^ within the junction space (*jnc*; [Ca^2+^_tot_]*_jnc_*) on the ordinate (upper) and the corresponding frequency is shown with the same parameter as in the upper panel on the abscissa (bottom); (**B**) a bifurcation diagram with the open probability of type II inositol 1,4,5-trisphosphate receptors (pO_InsP_3_R) on the abscissa and total amount of Ca^2+^ within the *jnc* ([Ca^2+^_tot_]*_jnc_*) on the ordinate (upper) and the corresponding frequency is shown with the same parameter as in the upper panel on the abscissa (bottom). Stable equilibrium points, unstable equilibrium points and stable limit cycles were plotted in red, black and green respectively in the upper panels of A and B; (**C**) results of time integration calculated using the same Ca^2+^ dynamics model as in (A). Ca_tot_ was changed as indicated in the lower panel in (**C**), and the spontaneous frequency was 0.04, 0.16, and 0.29 Hz at 4.18, 4.20, and 4.22 femtomole Ca_tot_, respectively. Note, the Ca^2+^ transient ([Ca^2+^]_blk_) is plotted in (**C**) instead of [Ca^2+^_tot_]_jnc_ in the bifurcation diagram. The [Ca^2+^]_jnc_ is the key factor initiating the spontaneous Ca^2+^ release.

Firstly, we fixed the total Ca^2+^ content (Ca_tot_) within a cell at a control level (3.20 femtomole) and found that the system was stable at a Ca_tot_ at junction space (*jnc*; [Ca^2+^_tot_]*_jnc_*) of 0.143 mM. The stable point was on the red curve on the left in Figure 1A (upper panel). Then, the amount of total Ca^2+^ was used as a bifurcation parameter and increased from 3.20 to 7.20 femtomole. The result shown in Figure 1A revealed that the system diverged at Ca_tot_ = 4.180 femtomole. When Ca_tot_ increased over the diversion point in the range of 4.180 to 5.923 femtomole, stable limit cycles (green) appeared with unstable equilibrium points (black). These results indicate that cyclic Ca^2+^ release occurred when Ca_tot_ was in the range plotted in green. In the usual numerical integration simulation, the peak and bottom level of the Ca^2+^ transients agreed well with the upper and lower level of the limit cycle depicted in green. As the α1-AR stimulation is mediated by the InsP_3_R, its open probability of the channel (pO_InsP3R_) was used as a bifurcation parameter in Figure 1B. Ca_tot_ was fixed at 3.6 femtomole for this analysis. At this low Ca_tot_ level, a stable equilibrium resting [Ca_tot_]*_jnc_* = 0.178 mM was observed. When pO_InsP3R_ increased, stable limit cycles were evoked between pO_InsP3R_ 0.0047 and 0.067. Increasing pO_InsP3R_ induced stable limit cycles at a Ca_tot_ level at which the system was stable without InsP_3_R activation, and vice versa, increasing Ca_tot_ induced stable limit cycles even when there was no InsP_3_R activation in this system. As is evident from the lower panels in Figure 1, the frequency of the automaticity increased markedly with increasing Ca_tot_ or pO_InsP3R_.

The connection between the limit cycle identified in the bifurcation analysis and the cycle of spontaneous [Ca^2+^] fluctuations in the bulk cytosol space (*blk*; [Ca^2+^]_blk_) was examined by conducting numerical time integration of the same minimal model (Figure 1C). Around the threshold Ca_tot_ level for initiating the limit cycle in Figure 1A, the Ca_tot_ was increased with a step size of 0.02 femtomole. The minimal model was quiescent up to 4.16 femtomole, and the cyclic [Ca^2+^]_blk_ transient was firstly observed at 4.18 femtomole. The frequencies of the Ca^2+^ transient increased with increasing Ca_tot_ as shown in Figure 1A. These responses of the minimal model were completely reversible when Ca_tot_ decreased.

### 2.2. Absence of Automaticity Inherent in Plasma Membrane Ionic Channels in the PVC

**Figure 2:**
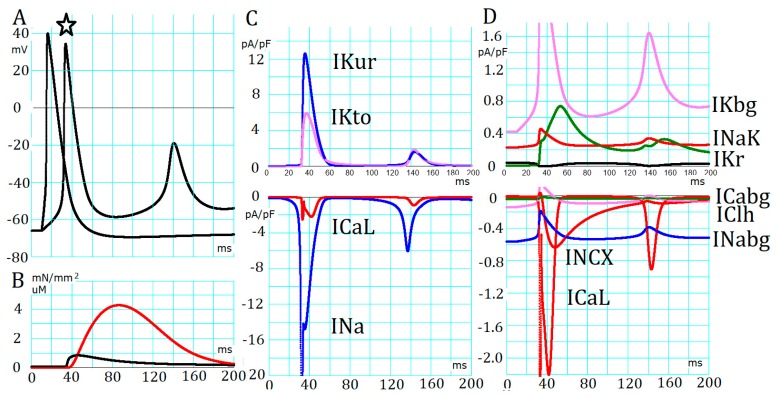
Membrane excitation in the absence of adrenergic (AR) stimulation: (**A**) Two records of action potential evoked by applying −5 (3 ms in duration) or –0.5 pA/pF (180 ms) current pulses were superimposed. The star mark indicates an action potential (AP) evoked with a smaller pulse; (**B**) the Ca^2+^-transient (black, μM) and the developed force of contraction (red, mN/mm^2^); (**C**,**D**) membrane currents shown at a low gain and at a high gain, respectively. Outward and inward currents are shown in the upper and the lower panels, respectively. Resting potential = −66.0 mV, [Na^+^]*_cyt_* = 5.7 mM, [K^+^]*_cyt_* = 117 mM, and [Ca^2+^]*_SRrl_* = 0.71 mM.

The PVC model showed a resting potential (V_rest_) at −68 mV, which was similar to that observed in experiments (>−75 mV). This less negative resting potential, compared to ventricular cells, is due to a much lower density of the inward rectifier potassium current (*I*_K1_) distribution compared with ventricular myocytes. Okamoto et al. revealed a hyperpolarization-activated Cl^–^ current (*I*_Clh_) at a voltage range more negative than the resting membrane potential (V_rest_) [8]. This *I*_Clh_ potentially stabilizes the low V_rest_ by antagonizing any kind of hyperpolarizing current, such as the *I*_K1_. *I*_Clh_ can provide the largest inward current during strong hyperpolarizing pulses, but at the V_rest_ level, the magnitude of *I*_Clh_ activation is the lowest in the present mathematical model (violet trace in Figure 2D lower panel). Although *I*_Clh_ was not calculated in most of the simulations, *I*_Clh_ potentially depolarized the V_rest_ by ~7 mV in the PVC model, as suggested in experiments by Okamoto et al. [8].

The membrane excitation was examined in a cell model after equilibration without application of electrical stimulation for ~100 s (Figure 2). The two records of action potential superimposed in Figure 2A were evoked by either a brief electrical stimulus (−5 pA/pF), or a longer and smaller pulse (190 ms in duration).

The AP duration was ~30 ms at −40 mV, which is in the same range of ventricular and atrial myocytes in rats [7,8,27], but is clearly shorter than AP in the SA node pacemaker cells in rats [28]. The maximum rate of AP increased was mediated by the transient component of sodium current (*I*_Na_) and was ~98.6 V/s. Repolarization was mainly caused by the inactivation of *I*_Na_, and was facilitated by the simultaneous activation of *I*_Kto_ and *I*_Kur_ (Figure 2C). The role of *I*_CaL_ in maintaining the plateau potential of cardiac AP was largely compromised by the much larger transient outward potassium current (*I*_Kto_) and ultra-rapid outward potassium current (*I*_Kur_). *I*_CaL_ triggered CICR from SR to evoke the peak amplitude of the Ca^2+^ transient of ~1 μM to induce the developed tension of the myofilaments (Figure 2B).

The record of membrane currents evoked by the long pulse are demonstrated in Figure 2C,D, which were used to examine membrane automaticity of the PVC. Depolarization to a less negative potential range than −60 mV evoked an AP, followed by brief damping oscillations, but failed to evoke repetitive APs. The V_m_ during the diastolic period is largely determined by the balance of major currents of outward-going *I*_NaK_, the background currents of *I*_Kbg_ and *I*_Kr_ (Figure 2D, upper panel), and the inward-going *I*_Nabg_ and *I*_NCX_ (Figure 2D, lower panel). Even if larger depolarizing current pulses were applied, no repetitive APs were observed. We concluded that the inactivation of *I*_Na_ during the AP was not removed at the diastolic potential levels during the current pulse because the activation of *I*_Kto_ and *I*_Kur_ quickly ceased during the falling phase of the AP. The amplitude of *I*_Kr_ (depicted in green in Figure 2D) is smaller than the *I*_Kbg_. This is in strong contrast to the pacemaker mechanism of SA node cells [28], where the *I*_CaL_ is relieved from inactivation through hyperpolarization caused by the activation of *I*_Kr_ by the preceding AP. The amplitude of *I*_Kr_ assumed in the present model (depicted in green in Figure 2D) was too small to generate an appreciable size of a tail current on repolarization, in agreement with the experimental study [8]. We concluded that no stable cycle of APs arises in the membrane ionic system of the PVC model.

### 2.3. Generation of AP_TD_s and an Activation Threshold for Ca^2+^_tot_

**Figure 3:**
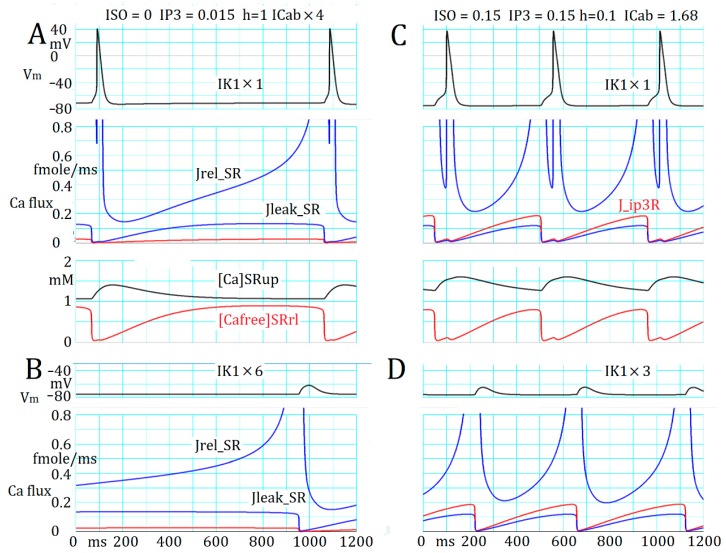
(**A**) Time-dependent changes in J_RyR_, J_IP3R_ (blue and red in the middle panel), [Ca^2+^]*_SRup_* and [Ca^2+^]*_SRrl_* (black and red in the bottom panel), when the repetitive action potential (AP; top panel) was evoked. (**B**) The transient depolarization (TD) when triggering APs was interfered with by increasing the inward rectifier potassium current (*I*_K1_) conductance six-fold. Almost the same time course of J_RyR_, [Ca^2+^]*_SRup_*, and [Ca^2+^]*_SRrl_* were observed but are not demonstrated. (**C**,**D**) Records were obtained as in A and B in the presence of noradrenaline (NA) stimulation. The triggering AP by the preceding TD in (**C**) was much delayed if compared with records in (**A**). This is because the amplitude of TD was partially depressed through the enlargement of *I*_NaK_ through an accumulation of [Na^+^]_i_ during the repetitive AP_TD_s.

The bifurcation analysis (Figure 1) applied to the intracellular Ca^2+^ dynamics in the absence of membrane ion fluxes revealed that spontaneous and repetitive Ca^2+^ transients were initiated by increasing Ca_tot_ above a certain threshold level (Ca_tot,thr_ = 4.18 femtomole) in the absence of plasma membrane ion fluxes. In the simulation shown in Figure 3, the spontaneous activity was recorded in the presence of intact plasma membrane currents.

The intracellular Ca_tot_ was technically increased step by step by enlarging the scaling factor of *I_Cabg_*. The spontaneous discharge of AP_TD_ appeared when Ca_tot_ was increased above 3.878 femtomole in the absence of AR stimulation, whereas the threshold Ca_tot_ decreased to 3.617 femtomole in the presence of 0.15 μM [ISO] and 0.15 μM [IP_3_]. Although the cycle length was markedly different, in both cases, the time course of V_m_ was flat during diastole except for a small slow diastolic depolarization of a few mV observed over ~300 ms after the preceding AP (Figure 3A,C). The Ca^2+^ flux (J_Ca_) from SR via RyR (passive leak component (J_leak_SR_) and active release component (J_rel_SR_)), and via InsP_3_R (J_InsP3R_) recovered in parallel with the replenishment of [Ca^2+^]*_SRrl_* within the following 400–500 ms because of the Ca^2+^ diffusion from SR uptake sites. The total continuous Ca^2+^ efflux (J_rel_SR_) finally evoked the final full Ca^2+^ release as indicated by the rapid fall of [Ca^2+^]*_SRrl_* through an increase in [Ca^2+^]*_jnc_*.

To confirm the involvement of TD in triggering APs, the amplitude of TD was depressed by increasing the membrane *I*_K1_ conductance temporarily by six-fold (Figure 3B) or by three-fold (Figure 3D) approximately 1–3 s before the start of the recorded segment in the figure. This intervention blocked the AP generation and revealed the presence of TD in response to the spontaneous Ca^2+^ release. The amplitude of TD was 10–13 mV and the *V_m_* at their peak was ca. –61 mV, most probably more negative than the threshold potential of *I*_Na_ activation.

Note that the downward deflection of [Ca^2+^]*_SRrl_* showed double peaks: One synchronized with the start of TD and the other with the foot of the AP (or the activation of *I*_CaL_) every time when AP_TD_ was triggered, whereas the second peak disappeared when TD failed to trigger AP_TD_.

The distribution of Ca_tot_ was 30% in SR uptake site (*SRup*), 24% in SR releasing site (*SRrl*), and 43% in *blk* mostly bound with the buffer, and the minor components were found in *jnc* (1.3%) and *iz* (2.3%) in control and remained within ±5% in various experimental conditions examined in the present study.

Alternatively, the AP_TD_s could be evoked by applying NA at 0.15 μM at the control *sfI*_Cabg_ = 1.68. Figure 3C,D show AP_TD_ as in Figure 3A,B, and demonstrate that the interval between the two successive AP_TD_s is much reduced (interval = ~500 ms), and the preceding TD evoked by the spontaneous Ca^2+^ release is obvious before the rising phase of the AP. Again, no slow diastolic depolarization was observed, and a rapid decay in *J*_Ca_ via a cluster of RyRs (couplon) clearly precedes the onset of TD, and the second notch of *J*_Ca_ was caused by the opening of LCC during the rising phase of the AP. The TD from the rising phase of the AP was isolated by increasing *sfI*_K1_ by three-fold (Figure 3D). The decrease in the TD amplitude in Figure 3D compared to that in Figure 3B was due to the enlarged *I*_NaK_ through the accumulation of [Na^+^] during the AP_TD_ burst.

The threshold Ca_tot_ level for initiating the AP_TD_ was examined in four combinations of two [ISO] of 0 and 0.2 μM, with two [IP_3_] of 0.015 and 0.15 μM. The threshold Ca_tot_ at the 0.15 μM [IP_3_] was higher (3.86 and 3.83 femtomole) than the value of (3.65 and 3.59 femtomole) obtained at 0.015 μM [IP_3_]. For a reference level of Ca_tot_, a representative 3.86 and 3.59 femtomole levels will be indicated in graphs of plotting time courses of Ca_tot_ in the following section.

### 2.4. Separation of α1- and β1-AR Influences on Ca_tot_ under Resting Condition

**Figure 4:**
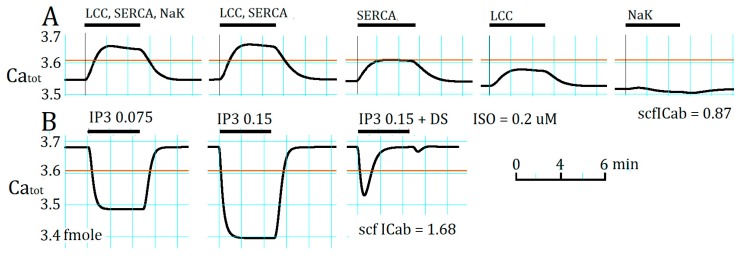
The influence of activating the individual components of NA-target molecules; (**A**) L-type Ca^2+^ channel (LCC), sarcoplasmic/endoplasmic reticulum calcium pump (SERCA), and Na^+^/K^+^ of the β1-AR; (**B**) InsP_3_R of the α1-AR. Ca_tot_ (femtomole) was plotted against the simulation time (min). The horizontal bars above the record indicate the duration of modification of each factor. The desensitization of the α1-AR was removed to determine the maximum influence of their influence on Ca_tot_, except in the last application of 0.15 mM [IP_3_]. The desensitization of the β1-AR was intact, but small in magnitude. The control level of Ca_tot_ was reduced in (**A**) by decreasing *scfI*_Cab_ to 0.87 to avoid a relatively large increase in J_Ca_SR_ evoked by increasing Ca_tot_ near the activation threshold in the absence of β1-AR stimulation. The horizontal thin brown line indicates the threshold level of Ca_tot_ in the presence of NA stimulation.

The findings in both Figure 1 and Figure 3 indicate that the increase in Ca_tot_ is the key factor for the initiation of the AP_TD_ in the present PVC model. Therefore, activation of the individual target components LCC, SERCA, and the Na^+^/K^+^ pump via β1-AR stimulation were examined by modifying Ca_tot_ at a saturating concentration of ISO (0.2 μM). In Figure 4A, the increase in Ca_tot_ evoked by the full member activation of β1-AR stimulation (LCC, SERCA, and Na^+^/K^+^ pump) was compared with different combinations of target activation. The increase in Ca_tot_ evoked by activating LCC plus SERCA was slightly larger than the control response. The separated Na^+^/K^+^ pump activation (the last trace in Figure 4) showed that the initial slight increase in Ca_tot_ was soon converted to a decrease during the ISO application due to a gradual decrease in Ca_tot_ through the NCX-mediated Ca^2+^ extrusion driven by the accelerated Na^+^/K^+^ pump. The influence of LCC activation is synergistic with the SERCA activation because these two molecules work together in transporting Ca^2+^ from extracellular space to the SR space. Ca_tot_ was markedly decreased by increasing the cytosolic [IP_3_] (Figure 4B), which was applied to represent the activation of α1-AR. The application of two concentrations of IP_3_ caused a dose-dependent decrease in Ca_tot_ as shown by removing the desensitization of the receptor at [IP_3_] of 0.075, and 0.15 μM. If the desensitization (DS) was intact (0.15 μM + DS), the initial decreasing phase of Ca_tot_ was interrupted by the time-dependent desensitization.

### 2.5. Marked Latency before the Onset of Repetitive AP_TD_ Generation after AR Stimulation

**Figure 5:**
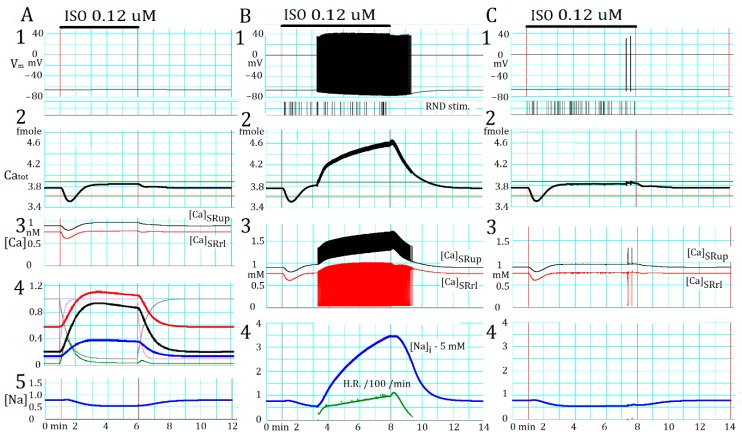
Initiation of a train of AP_TD_ by the α1-adrenergic receptor (AR) stimulation. The application of 0.12 μM ISO and 0.15 μM IP_3_ is indicated by the horizontal bars at the top of each column and the pair of vertical lines. (**A**) AP_TD_ was not induced. (**B**,**C**) The random triggering signals applied are shown in the lower part of B1 and C1 (random (RND) stim) to induce AP_TD_. The responses of V_m_ (1), Ca_tot_ (2), [Ca^2+^]_SRup_ and [Ca^2+^]_SRrl_ (3), and [Na^+^]_i_ (5 in **A** and 4 in **B**,**C**) are shown in each column. The time courses of *afI*_CaL_ (×0.5, red), *af*_SERCA_ (black), *af*_Na/K_ (blue), and pO_InsP3R_ (×100, green) shown in Figure 5(A4) are applicable to responses in (**B**,**C**). The rate of AP_TD_ discharge (/min/100) appears only in Figure 5(B4). The horizontal green and brown lines are the threshold Ca_tot_ measured in the absence and presence of AR stimulation, respectively.

In both tissue [9] and isolated rat cell preparations [7], a train of spontaneous AP was evoked after an extraordinary long latency (several minutes) after the application of NA. A plausible mechanism was already demonstrated in Figure 4B. The transient decrease in Ca_tot_ induced by activating InsP_3_R (α1-AR) might counteract the β1-AR activation, which promotes the AP_TD_ burst by increasing Ca_tot_ through *I*_CaL_ and SERCA activation (Figure 4A). This was the case, as shown in Figure 5(A2), where the Ca_tot_ rapidly decreased (by ~7%) after the start of AR stimulation. In this simulation, the control Ca_tot_ level was set slightly lower than the threshold of initiating AP_TD_ (green vertical line) in the absence of AR stimulation. Factors involved in this response are shown in Figure 5(A4). The open probability (pO_InsP3R_) of InsP_3_R (green) quickly increased from 0.00027 to a peak of 0.00385 and then decayed to 0.00039 due to the desensitization of the α1-AR receptor activation. The Ca_tot_ followed a time course similar to pO_InsP3R_ (Figure 5(A2)). The slightly higher desensitized pO_InsP3R_ than the control was due to a rise in Ca_tot_. Note, the InsP_3_R can be partially activated by Ca^2+^. Figure 5(A3) indicates that the [Ca^2+^]*_SRup_* and [Ca^2+^]*_SRrl_*, the major store site of Ca_tot_, followed the similar time course as Ca_tot_.

The time courses of the β1-AR stimulation of LCC (*af*_CaL_, red trace in Figure 5(A4)), SERCA (*af*_SERCA_, black), and Na^+^/K^+^ pump (*af*_NaK_, blue) are biphasic because of delayed fractional (30%) desensitization of β1-AR. The discharge of AP_TD_ evoked a full CICR, and thereby [Ca^2+^]*_SRrl_* decreased to a minimum level (Figure 5(B3)). The [Ca^2+^]*_SRup_* increased approximately parallel to the rise in [Ca^2+^]*_blk_*, and showed a transient increase due to the uptake of Ca^2+^ via SERCA at every Ca^2+^ transient. The wash out of the influence of AR stimulant took an exponential time course and lasted for ~2 min.

### 2.6. Involvement of the Spontaneous Membrane Fluctuations in Determining the Latency

In the control run shown in Figure 5A, the PVC remained quiescent because the Ca_tot_ level was still lower than the reference level (green line) of the spontaneous AP_TD_ discharge even after the desensitization of α1-AR. The AP burst was initiated only when the Ca_tot_ was further increased by augmenting the β1-AR stimulation. In such case, a train of AP_TD_s started at a given delay, different from the experimental finding of large variation in the 0–10 min latency [7]. For example, if *scfI*_Cabg_ was increased from 1.68 to 2.2, Ca_tot_ was still lower than the reference level, but the application of a saturating concentration of ISO (0.2 μM) evoked a train of AP_TD_ after a latency of 181.94 ± 0.5 s (mean ± SD, *n* = 13). This is in strong contrast to the marked experimental variations in the latency. We suggest variable time courses of gradual accumulation of Ca_tot_ after the AR stimulation. In the simulation (Figure 5(A2)), however, the accumulation of Ca_tot_ reached a peak within two minutes. We examined an alternative possibility of the miniature V_m_ fluctuations, most probably caused by sporadic CICR. The sporadic CICR was imitated using a random function (see Section 4) and the simulation results demonstrated in Figure 5B,C were obtained using the same protocol as in Figure 5A at the standard *sfI*_Cabg_ = 1.68 (Ca_tot_ = 3.765 femtomole). The frequency of sporadic CICR was approximately adjusted to the experimental records using a conventional RND function (see Section 4). The timing of random stimulation is shown by the vertical bars below the V_m_ record. Figure 5B,C provide typical examples of both success and failure of triggering repetitive AP_TD_s. The size of the sporadic CICR was variable at individual CICR events, as evidenced by the miniature fluctuations in [Ca^2+^]*_SRrl_* in the absence of continuous AP_TD_ generation (Figure 5C). Thus, the sporadic CICR mostly failed to evoke TD in visible amplitude to evoke AP_TD_, and the AP_TD_ was triggered randomly. In the case of Figure 5C, two sporadic AP_TDs_ were evoked toward to the end of the AR stimulation, but failed to start the repetitive AP_TD_s. In contrast, enough TD amplitude was evoked before the desensitization of β1-AR to trigger the AP_TD_ burst at a higher probability in Figure 5B.

The repetitive generation of AP_TD_s was progressively accelerated as indicated by the plot of discharge frequency (Figure 5(B4), green curve). This is consistent with the bifurcation analysis results (Figure 1) that the frequency of the limit cycle of events increased with increasing Ca_tot_ in the Ca^2+^ dynamics when separated from the membrane function. In Figure 5B, the discharge of AP_TD_ increased Ca_tot_ through the activation of membrane *I*_CaL_, and thereby positive feedback is involved in the rising phase of the spontaneous rate. This positive feedback was counteracted by the progressive increase of outward-going *I*_NaK_ through the accumulation of [Na^+^]_cyt_ (Figure 5(B4)), which was induced by the Na^+^/Ca^2+^ exchange via NCX. If the accumulation of [Na^+^]_cyt_ was augmented by increasing the β1-AR simulation, the increase in outward *I*_NaK_ current blocked triggering AP_TD_ by decreasing the amplitude of TD (not shown). In this case, the repetitive AP_TD_ generation was interrupted, but resumed when the accumulation of [Na^+^]_cyt_ was relieved by the extrusion of Ca^2+^ via NCX. Thus, the AP_TD_ burst occurred intermittently (not shown). Note, [Na^+^]_cyt_ was decreased via augmentation of the Na^+^/K^+^ pump by the β1-AR stimulation when the repetitive AP_TD_s were absent (Figure 5A,C).

### 2.7. Latency Histogram of the AP_TD_ Burst

**Figure 6:**
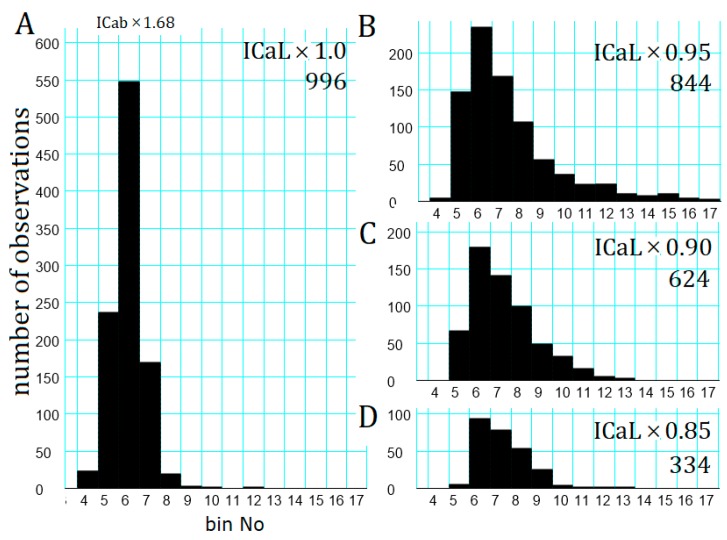
Latency histogram of the initiation of repetitive AP_TD_ generation at different amplitudes of *I*_CaL_. The latency was measured from the onset of NA application to the time of the initial fifth AP_TD_ discharge within a train. Vertical and horizontal axes for (**B**–**D**) are the same as in (**A**). The bin width was 30 s. The amplitude of *I*_CaL_ in each series of simulation is indicated at up-right corner of (**A**–**D**) together with the number of successful triggerings of the repetitive generation of AP_TD_s in 1000 trials.

The latency was measured by constructing a latency histogram from several data sets of 1000 trials of the AR stimulation, as shown in Figure 6, where each data set was obtained at the control Ca_tot_ (*scfI*_Cab_ = 1.68) but at different amplitudes of *I*_CaL_. In the control run (*I*_CaL_ × 1.0, Figure 6A), the burst was successfully evoked in 996 trials, and the histogram showed a peak at bin No. 6 of 2.5–3.0 min. The success rate evoking a repetitive AP_TD_s was sensitive to the amplitude of *I*_CaL_ (Figure 6B–D), and the success rate drastically decreased to 33.4% by reducing the amplitude of *I*_CaL_ by 15% (Figure 6D). This success rate is near to that (26.7%) obtained in the experimental study [7]. The size of Ca^2+^ influx due to the activation of *I*_CaL_ and the uptake of Ca^2+^ into SR by SERCA play a key role in determining the time-dependent increase in Ca_tot_. Note, the [Ca^2+^]*_nd_* is largely dependent on [Ca^2+^]*_SRrl_* when a continuous Ca^2+^ leak is generated by both InsP_3_Rs and leak conductance of couplons. 

## 3. Discussion

### 3.1. Brief Summary 

**Figure 7:**
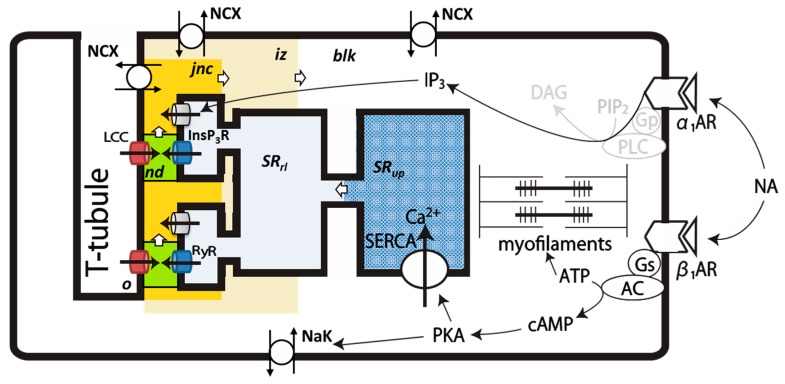
Signal transduction pathways for the α1- and β1-ARs, intracellular Ca^2+^ compartments, and distribution of Ca^2+^-transporting channels and transporters. Components in gray are not included in the model. Open arrows between compartments indicate the usual diffusion paths of Ca^2+^. The activation of type II ryanodine receptors (RyRs) and inactivation of LCC are determined by [Ca^2+^]*_nd_*. The Ca^2+^ flux through InsP_3_R directs to *jnc.* If both LCC and RyRs are closed, [Ca^2+^]*_nd_* equals [Ca^2+^]*_jnc_*.

The PVC model developed in the present study reconstructed well the electrical activity recorded in the PVCs dissociated from rat pulmonary veins by Okamoto et al. [7,8]. The model structure of CICR was refined in the human ventricular cell model [16], and was adopted in the presented PVC model including the Ca^2+^ spaces in the cytosol (Figure 7). The initiation of spontaneous APs in the model was attributed to the CICR (Figure 3), which was enhanced by the NA application, whereas the intrinsic membrane ionic mechanisms, described in cardiac pacemaker cells are not sufficiently developed to trigger spontaneous APs (Figure 2). After all membrane ionic fluxes were removed, the bifurcation analyses disclosed stable limit cycles over a certain range of Ca_tot_ within the cell (Figure 1). The threshold level for the initiation of the repetitive AP_TD_ generation was slightly lower in the full model than in the model of cytosolic Ca^2+^ dynamics (Figure 5), most probably because the membrane components, such as NCX and LCC, which enhanced the positive feedback mechanisms of the spontaneous rhythm, are not involved in the minimal model. We demonstrated that the β1-AR stimulation increased Ca_tot_, whereas the α1-AR activation temporary decreased Ca_tot_ (Figure 4). The marked latency of several minutes for the start of repetitive AP_TD_ generation after the onset of stimulation could be explained by assuming the desensitization of α1-AR (Figure 5). Since the experimental findings on both the intracellular Ca^2+^ dynamics and signal transduction in rat PVC cells are still limited, the model proposed in this study may be revised in future studies. The presented simulation model, however, should provide a useful working hypothesis for conducting new experiments using dissociated cells.

### 3.2. Co-Localization of InsP_3_R with RyRs in the Sub-Sarcolemmal Space Supporting Spontaneous CICR

The experimental studies in rat atrial myocytes demonstrated that a long lasting Ca^2+^ release from SR is induced by IP_3_, and through the co-localization of InsP_3_R with RyRs, the transient Ca^2+^ release is evoked [10]. Essentially the same mechanism was demonstrated when the InsP_3_R was activated by endothelin-1 (ET-1) [11]. The Ca^2+^ extrusion through NCX is augmented by this transient increase in [Ca^2+^] and thereby a temporal transient membrane depolarization is evoked due to the electrogenic stoichiomety of Na^+^/Ca^2+^ exchange [19,20,21,22]. Mackenzie et al. directly recorded Ca^2+^ sparks as well as premature APs in the presence of ET-1 at a high probability of 75% in trials in rat atrial myocytes under α1-AR stimulation [11]. They demonstrated that a TD was evoked by an overlap of multiple Ca^2+^ sparks. The involvement of InsP_3_R was proven by recording larger amplitudes of premature AP when a membrane-permeable analogue of IP_3_ was applied in the extracellular medium [10]. The results of this simulation study using a new mathematical model of PVC in the present study agree with the basal Ca^2+^ mechanisms suggested by the previous studies. In the present study, a functional coupling of InsP_3_R with RyRs was achieved by connecting the Ca^2+^ flux through InsP_3_R to the hypothetical *jnc*, which directly encircles multiple CaRUs based on nanodomain (*nd*), consists of LCC and the couplon. Note that in the absense of LCC activation at the resting membrane potential, the spontaneous activation of a couplon is only controlled by the [Ca^2+^] within this *jnc* in the Hinch formalism of CaRU [14,15,16].

### 3.3. Peculiarity of AP_TD_ Generated in PVCs Compared to Aatrial and Ventricular Myocytes

The spontaneous TD and the AP_TD_ have been observed by activating the α1-AR more frequently in the atrial than in ventricular myocytes [10]. Atrial myocytes express InsP_3_R at a much higher density than in the ventricular myocytes. InsP_3_Rs are co-localized with the couplons (RyRs) in the junctional SR, but are absent in the network SR (non-junctional SR) in the atrial myocytes. The T-tubules are densely distributed at each interval of the sarcomere pattern, and thereby the sum of the T-tubule membrane is almost comparable to the surface membrane in ventricular myocytes (~78%) [29]. Thus, the junctional SR is distributed throughout the depth of the myocytes. However, the ratio of InsP_3_R-coupled couplons to non-coupled couplons should be much lower in the ventricular myocytes. Thus, the spontaneous CICR may occur at a much lower frequency in the ventricular myocytes.

The PVCs show T-tubules and the InsP_3_R distributed along the sarcomere pattern as in the ventricular myocytes [7]. Thus, it is expected that the density of couplons co-localized with InsP_3_R should be much higher in PVCs than in atrial myocytes. This well agrees with PVs showing high frequencies of AP_TD_s in both tissue preparations [2] as well as in dissociated cardiomyocytes [7], differently from the sporadic discharge of AP in the atrial myocytes during the activation of InsP_3_R [10,11]. Note, the high frequency of AP_TD_ generation is attributable to the frequency of the intracellular Ca^2+^ oscillations as indicated by the bifurcation analysis (Figure 1). The low resting membrane potential (−70 mV) with low membrane conductance, most probably due to the low density of the *I*_K1_ distribution, may further facilitate the discharge of repetitive AP_TD_s. In conclusion, The AP_TD_s may be evoked by essentially the same mechanism during AR stimulation as in the other working myocardial cells. The mechanisms may be highly enhanced in the PVCs to generate spontaneous repetitive AP_TD_s in rat.

### 3.4. Ca^2+^ Overload and β1-AR Stimulation Evoking Repetitive AP_TD_ Generation in PVCs

Okamoto et al. observed that spontaneous TDs or miniature potential fluctuations occurred at the resting potential even before the application of NA [7]. These potential fluctuations are barely observed in the working myocytes, such as atrial or ventricular myocytes, but was induced with a high success rate by depressing the Na^+^/K^+^ pump by applying digitalis. This response is explained by the “Ca overload”, which was expected to occur secondarily to the increase in [Na^+^]_i_ [19,22]. The Ca^2+^ overload is also caused by the repetitive stimulation of ventricular myocytes in the presence of α1-NA stimulation, and the spontaneous Ca^2+^-release from SR evokes the TD [30]. We introduced a hypothetical *I*_Cabg_ for the purpose of varying the Ca_tot_ in PVCs. In the present study, the threshold of the AP_TD_ discharge was determined by slowly increasing Ca_tot_ in a stepwise manner to allow for a quasi-steady state distribution of Ca^2+^ within the cell in each step change. The threshold Ca_tot_ levels of 3.88, 3.87, 3.66, and 3.61 femtomole were determined in the control, α1-AR, β1-AR, and α1- plus β1-AR stimulation, respectively, after removing the desensitization of both receptors. This result is in good agreement with the finding in Figure 1 that the limit cycle of Ca^2+^ release was evoked simply by increasing the Ca_tot_ in the absence of the membrane ionic mechanisms.

### 3.5. The Latency and Frequency of AP_TD_s Under NA Effects

The simulation results in Figure 5 strongly suggest that the latency before the initiation of the AP_TD_ generation might be determined by the desensitization of α1-AR in PVCs. In general, G_q_-coupled receptors, such as α1-AR, ET-1 receptor, and angiotensin receptors, show desensitization after the binding of ligands [31,32,33]. The time course of desensitization has been demonstrated for the ET-1 and angiotensin receptors [34,35]. Cooling et al. [17] successfully developed a mathematical model of the signal transduction evoked by the G_q_-coupled receptors according to the experimental measurements. They suggested that the time course of desensitization is largely determined at the level of receptor molecules. However, they did not discuss the desensitization time course of α1-AR. We failed to find any experimental measurements of the α1-AR desensitization time course in cardiac myocytes. We only observed several references in which indirect findings were described without interpretation by the authors. Zhang et al. recorded the *I*_CaL_ response to phentolamine application in rat ventricular myocytes, and found that the *I*_CaL_ amplitude temporarily decreased over several minutes before the gradual increase in *I*_CaL_ [36]. They suggested that the decrease might be due to Ca^2+^-mediated inactivation induced by a temporal release of SR Ca^2+^ through InsP_3_R. Terzic et al. observed that the cell shortening evoked by the electrical stimulation of rat ventricular myocytes temporarily decreased in the same time course as *I*_CaL_ [37]. They suggested that this decrease in shortening might be due to the depletion of Ca^2+^ in SR. These findings strongly suggest the desensitization of α1-AR during phentolamine application. Although these responses were conducted at different ambient temperatures, the time courses were rather similar to the desensitization time course of α1-AR assumed in the present study.

In general, the subthreshold TDs appeared randomly as described in atrial myocytes [10,11]. The variation in the latent period before the initiation of the AP_TD_ generation is in agreement with the random occurrence of subthreshold TDs accompanied by transient elevations in intracellular Ca^2+^ [7]. To simulate this sporadic triggering of AP_TD_, the regularity of spontaneous generation of AP_TD_ burst was avoided by lowering the Ca_tot_ to below the threshold level in the presence of β1-AR activation. Then, the train of AP_TD_ was triggered by the hypothetical random CICR. The experimental histogram obtained by Okamoto et al. [7] showed events of shorter latency than the time span of desensitization (~1.5 min). These shorter latency events might be simulated by increasing the conditioning Ca_tot_, or by decreasing the conductance of whole-cell InsP_3_R. In experiments, these parameters might be largely variable between individual PVCs, so that the latency histogram showed a wider distribution in the experimental study than in the present simulation study, in which the histogram was obtained using one cell model with a set of initial values of the variables.

### 3.6. Coupling Several Layers of Physiological Mechanisms to Regulate Ca_tot_ in PVCs

A regular time course of [Ca^2+^]*_SRrl_* change (core cycle) is evoked when the cell is stimulated by an isolated electrical stimulus; the CICR transiently depletes [Ca^2+^]*_SRrl_* at the terminal cysterna through excitation-contraction (EC) coupling. Then, the [Ca^2+^]*_SRrl_* gradually recovers through the Ca^2+^ diffusion from the Ca^2+^ uptake site of SR. If Ca^2+^ leaks through RyRs and InsP_3_R are added to this core cycle, any Ca^2+^ accumulation in *jnc* potentially triggers the next event of Ca^2+^ release via CaRU. The bifurcation analysis (Figure 1) indicated that a stable limit cycle of this Ca^2+^ release is evoked by increasing the Ca_tot_ beyond a certain level in the absence of Ca^2+^ flux across the cell membrane.

If the membrane ionic fluxes are coupled with the core cycle of Ca^2+^ oscillation, positive and negative feedback mechanisms are added. As a negative feedback, the transient Ca^2+^ release via couplons primarily accelerates extrusion of Ca^2+^ to the extracellular space via NCX. If an enlarged TD, evoked by the electrogenic Na^+^/Ca^2+^ exchange, triggers an AP_TD_, the extra Ca^2+^ influx through LCC activation can increase Ca_tot_, which may accelerate the core cycle of Ca^2+^ fluctuation and the spontaneous discharge of AP_TD_ to start the positive feedback cycle to further augment the Ca_tot_. 

The extrinsic regulation Ca_tot_ via β1-AR and α1-AR activation may further potentiate finer but complicated tuning of the Ca^2+^ dynamics. Basically, the simultaneous activation of both LCC and SERCA efficiently accumulates Ca_tot_ through Ca^2+^ uptake into SR. The activation of InsP_3_R through α1-AR activation strongly decreases Ca_tot_ in combination with the Ca^2+^ extrusion by NCX. This simple antagonistic relationship between α1- and β1-ARs can be reversed to a synergistic relationship through the mechanism where by the frequency of the repetitive AP_TD_ generation is markedly increased by the faster recovery of [Ca^2+^]*_SRrl_* by facilitating positive feedback.

So far, the influences of AR stimulation revealed in the present study were mostly suggested under the maximized strength of both α1- and β1-AR stimulation. Therefore, the mechanisms revealed by such pathophysiological simulations might not be applicable to the homeostatic regulation of Ca_tot_ under physiological conditions, where a much finer neural regulation may be expected. For example, the neural activity of peripheral sympathetic fiber always changes dynamically by repeating short bursts of APs in synchrony with fluctuations in arterial pressure [38]. Under such moderate stimulation, a vast desensitization, as observed in Figure 5 or in real experiments during the continuous stimulation for ~10 min, might not be expected. Most probably, the α1-AR stimulation may simply stabilize the positive inotropic influence through the β1-AR stimulation. The enhancement of Na^+^/K^+^ pump via β1-AR stimulation as well as the InsP_3_R activation via α1-AR should compromise the increase in Ca_tot_ through an enhancement of NCX. The conduction of the AP from the atrial tissue may induce stable positive inotropy of PVCs through the β1-AR stimulation.

However, the simulation of the experimental findings successfully demonstrated cellular mechanisms in the present study. Thus, the mathematical model proposed is feasible for further examination of physiological mechanisms of the PVCs or might be applied to other cell types.

### 3.7. Limitations

The functional mechanisms of individual components of the presented PVC model had not yet been clarified by conducting experiments in PVCs. To overcome this issue, the components of the model were developed based on the general reaction scheme, which has been established in a variety of cardiac cell types. This situation of limited experimental data is similar to that of developing human cardiac myocytes. However, mathematical models of human cardiac myocytes have been useful in providing new working hypotheses. The PVC model provided an assumption that the relatively long latency of several minutes should be proved by directly examining the desensitization of α1-AR. Variations in Ca_tot_ should be estimated or considered when the time course of NA effect is examined.

Experiments in both tissue and dissociated myocytes demonstrated that the AP_TD_ burst was terminated with a long latency of many minutes after selectively blocking the α1-AR [2,7,9]. However, our simulation study failed to reconstruct this finding because the α1-AR was already desensitized to the control level in the presented simulation before the application of the α1-AR blocker. To address this issue, the involvement of other factors should be considered, such as the activation of *I_CaL_* by the α1-AR pathway and/or a decrease in membrane K^+^ conductance, which takes a relatively long time course to develop. The nature of membrane K^+^ conductance (*I*_Kbg_) depressed through α1-AR stimulation has not yet been clarified. Alternatively, the desensitization of α1-AR might partially occur and some fraction remains intact. If so, the pharmacologic blockade of α1-AR might decrease the frequency of repetitive AP_TD_ generation as indicated by the relationship between InsP_3_R conductance and the frequency (Figure 1); thereby, the positive feedback cycle between the increase in spontaneous frequency of AP_TD_ through the accumulation of Ca_tot_ might be blocked, resulting in the cessation of spontaneous activity. We could simulate this mechanism under some narrow spectrum of the combination of modifying Ca_tot_, degree of desensitization, or the decrease in the membrane K^+^ conductance. The complex mechanisms underlying the pluripotent nature of α1-AR stimulation remain to be clarified [36,37,39,40,41,42,43,44,45,46].

## 4. Materials and Methods 

### 4.1. Intracellular Ca^2+^ Compartments and Distribution of Ionic Channels and Transporters in the PVC Model

Figure 7 shows a schematic representation of the model structure of a rat PVC. The T-tubule membrane provides the counterpart of the dyadic junction for the CICR. According to the Hinch model of the CaRU [14,15], individual CaRU is composed of one or a few number of LCC on the T-tubule membrane facing the couplons (clusters of RyRs) on the SR membrane. For activation and inactivation, both couplons and LCC refer to the same local Ca^2+^ concentration in the nanodomain (*nd*) cleft depicted in green. Each CaRU is separated from the others to support the local control of CICR, but a moderate cooperativity of multiple CaRUs is conserved by a local Ca^2+^ domain called *jnc*, which allows a temporal accumulation of released Ca^2+^ as firstly described in the HuVEC model [16,47]. Ca^2+^ accumulated in *jnc* gradually diffuses to *iz* and then to *blk*, in which myofilaments are located. Increased Ca^2+^ in each compartment is partly extruded by NCX. Note, only two representative CaRUs are demonstrated among many numbers of CaRUs in Figure 7. All CaRUs share a single common Ca^2+^ uptake site of the network SR spread in the *blk* for computational simplicity.

The major compartment of SR is *SR_up_* equipped with SERCA, and provides its extension (terminal cysterna) to form *SR_rl_* as the other counterpart of the dyadic junction. The *SR_rl_* site contains the Ca^2+^ binding protein, calsequestrin, to increase the capacity of Ca^2+^ release. The Ca^2+^ released via InsP_3_R diffuses into the *jnc*. The ion channels and transporters are exposed to different [Ca^2+^] after the Ca^2+^ release, and their distribution to each compartment is given in the Supplemental Materials, but not shown in Figure 7. During the Ca^2+^ release, [Ca^2+^]*_SRrl_* is depleted to halt the Ca^2+^ release through the couplons.

NCX is distributed to *jnc*, *iz*, and *blk*, and their activation evokes TD. If the peak of transient depolarization crosses the activation voltage threshold of *I*_Na_, AP_TD_ is triggered. A series of AP discharge occurs when Ca_tot_ within the cell is increased above a threshold level. In the present study, the extent of Ca^2+^ overload was adjusted simply by magnifying the hypothetical *I*_Cabg_ assumed on the surface membrane. Simulating various mechanisms of the Ca^2+^ overload was beyond the scope of the present study. The myofilament contraction model of Negroni and Lascano (2008) was adopted to calculate the free Ca^2+^ concentration in the *blk*.

### 4.2. Relationship Between Local [Ca^2+^]_nd_ and Spontaneous Ca^2+^ Release

In the Hinch model, the [Ca^2+^]_nd_ is determined in different combinations of LCC and couplons under the assumption of instantaneous Ca^2+^ distribution [14,15].

(1) $${\left[Ca^{2+}\right]}_{nd}=\frac{{\left[Ca^{2+}\right]}_{jnc}+f_{R}\times {\left[Ca^{2+}\right]}_{SRrl}+f_{L}\times \frac{\delta V\cdot e^{-\delta V}}{1-e^{-\delta V}}\times {\left[Ca^{2+}\right]}_{o}}{1+f_{R}+f_{L}\times \frac{\delta V}{1-e^{-\delta V}}},\;f_{R}=0.31,\;f_{L}=0.014.$$

The opening of LCC or a couplon is approximately represented by the parameters *f_R_* and *f_L_* defined by Equations (2) and (3), respectively. *f_L_* and *f_R_* are zero when LCC and couplons are closed, respectively.

(2) $$f_{R}=\frac{G_{R}}{G_{diff}},$$

(3) $$f_{L}=\frac{G_{L}}{G_{diff}},$$

where *G_L_* and *G_R_* are Ca^2+^ conductivity through the LCC and couplons, respectively; and *G_diff_* represents the conductivity of Ca^2+^ diffusion across the hypothetical border between *nd* and *jnc*. In the case of couplons, its activation is assumed to occur in an all or none manner; thus, *G_R_* represents the limiting conductance of the couplon, and *f_R_* provides the proportional coefficient in respect to [Ca^2+^]*_SRrl_*. In the case of LCC, a single LCC is assumed so that the conductance G_L_ represents the limiting conductance of LCC, but is an exponential function of the membrane potential V_m_ as described by Equation (1). Note, *f_L_* and *f_R_* also provide proportional coefficients for [Ca^2+^]*_nd_* in respect to [Ca^2+^ ]*_o_* in the extra-cellular solution and [Ca^2+^]*_SRrl_*, respectively.

An increase in [Ca^2+^]*_nd_* is the sole factor in determining the spontaneous Ca^2+^ release via couplon. Then, [Ca^2+^]*_jnc_* is calculated from the total amount of Ca^2+^ within the space ([Ca_tot_]*_jnc_*), which is determined by the four Ca^2+^ fluxes as represented by Equation (4).

(4) $$\frac{d{\left[Ca_{tot}\right]}_{jnc}}{dt}=-\frac{J_{Ca,m}+J_{Ca\_couplon}+J_{Ca\_InsP3R}-J_{Ca\_jnciz}}{V_{jnc}},$$

where *V_jnc_* is the volume of *jnc*, and *J*_Ca,m_, *J*_Ca-couplon_, *J*_Ca-InsP3R_, and *J*_Ca,jnciz_ are membrane Ca^2+^ fluxes in the *jnc* space, Ca^2+^ release through couplon and InsP_3_R, and Ca^2+^ diffusion between the *jnc* and *iz*, respectively. Under a Ca^2+^-overload condition, the J_Ca_rel_ through the basal openings of the couplon or activated InsP_3_R increases in proportion to the level of [Ca^2+^]*_SRrl_* above the threshold level. The amplitude of Ca^2+^flux is determined by Equations (5) and (6).

(5) $$J_{Ca,RyR}=G_{RyR,bg}\cdot ({\left[Ca^{2+}\right]}_{SRrl}-{\left[Ca^{2+}\right]}_{jnc}),$$

(6) $$J_{Ca,IP3R}=G_{IP3R,bg}\cdot ({\left[Ca\right]}_{SRrl}-{\left[Ca\right]}_{jnc}).$$

For Ca-buffers in *jnc*, *iz*, and *blk*, see Supplemental Materials. 

### 4.3. Ion Channels and Transporters

All ion channels and transporters included in the present PVC model are listed with references in the Abbreviations section. All the ion channels and transporters are distributed in each Ca^2+^ compartment (Table 1).

The Cl^−^ channel having a large conductance was described during hyperpolarization in the rat PVC cells [8]. However, including *I*_Clh_ in the PVC model is difficult, since no experimental report on Cl^−^ transporters is available for the PVC cell. To obtain Cl^−^ homeostasis, we need to balance the passive Cl^−^ flux with some Cl^−^ transporters. At present, the PVC model does not include *I*_Clh_. In preliminary simulations, *I*_Clh_ was included only to estimate its contribution to the membrane potential. We found that the contribution of *I*_Clh_ to the membrane potential is relatively small at V_m_ less negative than −70 mV.

The equations of these ion currents and the transporters are given in Supplemental Materials. Only several currents with immediate significance for the spontaneous APD_TD_ are described below. 

In rat PVC cells, the amplitude of *I*_Na_ was larger when compared with *I*_Na_ in the atrial tissue, and the activation range was shifted to left in the current–voltage (I–V) relationship [6,48]. No obvious difference was reported in the density and kinetics between PVC and LA [3]. Okamoto et al. demonstrated that the inactivation of *I*_CaL_ is faster in the rat PVC cells compared to ventricular cells [7]. In the CaRU of the HuVEC model, the locus of Ca^2+^-mediated inactivation of LCC is *nd* and the inactivation rate (k_oc_) is described as:

(7) $$k_{oc}=\frac{{\left[Ca^{2+}\right]}_{x}}{K_{L}}\cdot \frac{fV_{m,act}}{T_{L}}\;K_{L}=0.0044\;mM,\;T_{L}=147.51\;,$$

where *fV_m,act_* is a parameter for the V_m_-dependent activation parameter and is used to describe the dependence of LCC inactivation on the opening of the voltage gate, and *K_L_* is used to represent the [Ca^2+^]-dependence in *nd* or other [Ca^2+^]. We assumed that 75% of *I*_CaL_ was connected to *jnc*, and 15% to *iz*, and the rest to *blk*. A slightly smaller *K_L_* (*K_L_* = 0.00154 mM) was used for the at PVC cell model to increase the Ca^2+^ sensitivity.

Virtually no obvious amplitude of *I*_Kr_ tail current has been recorded on the jump from the positive potential to the holding potential in the voltage clamp experiment in rat PVCs [7]. However, in rat atrial cells [49,50], ventricular cells [50], and canine PVCs [3], the existence of *I*_Kr_ was revealed at a relatively small density. We included a minimum size of *I*_Kr_ in the PVC model to provide a repolarization reserve for technical reasons to avoid instability of V_m_, which was observed around –20 to 40 mV after the inactivation of both *I*_Kto_ and *I*_Kur_. Activation of a transient outward current was observed at the onset of the depolarizing pulse in the rat ventricular myocyte model by Pandit et al. [51]. The *I*_Kur_ model was adopted from the mouse ventricular myocyte model by Bondarenko et al. [52].

The *I*_Clh_, found by Okamoto et al., showed slow voltage-dependent activation on hyperpolarization with a time course similar to that of the hyperpolarization-activated non-selective cation current in the SA node cell [8]. Thus, we applied the kinetic equation of *I*_h_ simplified by reducing the number of states to C_1_, C_2_, and O states after optimizing the rate constants. The *I*_K1_ in PVCs seems much smaller in amplitude compared to the ventricular cells to allow the relatively low resting potential in PVC cells [7].

The histochemical examination of the NCX by Okamoto et al. [7] disclosed the localization of NCX near the T-tubules. This finding was represented by the 28% distribution of NCX near the T-tubule (2% in *jnc* and 25% in *iz*) and the rest in *blk*, respectively (Table 1).

### 4.4. Simulation of NA Stimulation of PVCs 

The mathematical model of the β1-AR stimulation developed by Saucerman et al. [18] was adopted after minor modification [53,54]. The reaction cascade that generates cytosolic cyclic AMP (cAMP) and the activation of PKA evoked by β1-AR agonist ISO is described in the Supplemental Materials.

Doisne et al. never observed spontaneous activity in isolated pulmonary veins of rat under basal physiological conditions, but observed that the application of norepinephrine induced automatic electric activity [9], in agreement with the contractile activity observed by Maupoil et al. [2]. Okamoto et al. observed the spontaneous repetitive AP generation when α1- and β1-ARs were stimulated in dissociated PVCs, and also recorded major ionic current components of the PVCs [7]. In reconstructing the experimental data of Okamoto et al. [7,8], we selected SERCA, LCC, and the Na^+^/K^+^ pump as key targets of the β1-adrenergic regulation, and InsP_3_R and a kind of background K^+^ conductance for the α1-AR regulation. Although RyRs are also the target of the β1-adrenergic regulation, we did not calculate the modification of RyRs for simplicity, partly because the activation of the couplon occurred in an all-or-none manner during the CICR. Here, the “catecholamine effect” denotes the sum of these effects. The influence of individual affecters were examined by varying the activity of the corresponding target molecule.

#### 4.4.1. Implementation of β1-AR Effects

We assumed two populations of the target molecules for each of Na^+^/K^+^ pump, SERCA, and LCC; one is the phosphorylated fraction (*Fr_PKA_*) by the catalytic subunit of PKA and the other is the dephosphorylated fraction by several kinds of phosphatase (PPs). In a normalized scheme, the reaction of phosphorylation is described by Equation (8):

(8) $$(1-Fr_{PKA})\begin{array}{c} \overset{\alpha }{\to }\\ \underset{\beta }{\leftarrow } \end{array}Fr_{PKA}.$$

The time course of *Fr_PKA_* after the onset of β1-AR stimulation was calculated using Equation (9).

(9) $$\frac{dFr_{PKA}}{dt}=\alpha \cdot (1-Fr_{PKA})-\beta \cdot Fr_{PKA},$$

where a common time constant (τ) of phosphorylation was assumed for the three kinds of target protein. Since the activation time course takes several tens of seconds after the application of β1-AR stimulant, a τ of 40 s was assumed for convenience. The forward rate α in Equation (8) is dependent on the concentration of catalytic subunit of PKA (*cat*), a forward rate constant *k_cat_* = 0.0625/mM/ms was determined by model adjustment, and the backward rate constant β was obtained by Equations (10) and (11).

(10) $$\alpha =k_{cat}\cdot cat,$$

(11) $$\beta =\frac{1}{\tau }-\alpha .$$

When a basal phosphorylation (*base*) of target protein caused by kinases other than PKA is assumed, the sum of phosphorylated active fraction (*af*_(t)_) of a target protein is given as a sum of two components denoted as *base* and *delta*.

(12) $$af(t)=base+delta\cdot Fr_{PKA(t)}.$$

The proportion of base/(base + delta) for Na^+^/K^+^ pump, SERCA, and LCC were model adjusted to 25%, 10%, and 43.5%, respectively.

#### 4.4.2. *Na^+^/K^+^ Pump*

The Na^+^/K^+^ pump model used in the human ventricular cell (HuVEC) model [55] was adopted, since it was developed by referring to a wide variety of electrophysiological findings to apply the detailed thermodynamic framework of the original Na^+^/K^+^ pump model [56]. The β1-adrenergic regulation of the Na^+^/K^+^ pump model was introduced by implementing the involvement of phospholemman (PLM) as described by Despa et al. [57]. According to their study, the activation of Na^+^/K^+^ pump by the phosphorylation of PLM was represented by a decrease to 75% the control in the apparent Na^+^-dissociation constant (*Kd_,Nai_*) of the cytosolic binding site. The decrease was calculated by applying a scaling factor *sf* to the original *K_d_* in the four state model of Na^+^/K^+^ pump.

(13) $${\bar{Kd}}_{Nai}=sf_{NaK}\cdot Kd_{Nai}.$$

To satisfy the thermodynamic constraint of:

(14) $$\frac{k_{1}^{+}k_{2}^{+}k_{3}^{+}k_{4}^{+}K_{d,K_{i}}^{2}K_{d.Na_{e}}^{3}}{k_{1}^{-}k_{2}^{-}k_{3}^{-}k_{4}^{-}K_{d,MgATP}K_{d,K_{e}}^{2}K_{d.Na_{i}}^{3}}=K_{\sim MgATP}\cdot e^{FV_{m}/RT},$$

where the *Kd,_Ke_* for the extracellular binding site was increased by *sf*^−3/2^ to obtain the increase in the I_NaK_ [55,57], a *sf*_NaK_ = 0.72 was set for the Na^+^/K^+^ pump relieved from the inhibitory action of PLM.

We assumed two populations of the Na^+^/K^+^ pump, with phosphorylated PLM fractions (activated fraction, *af*) and non-phosphorylated fraction (1 – *af*). The whole cell *I*_NaK_ was determined as a sum of control *I*_NaK_ and activated ${\bar{I}}_{NaK}$,

(15) $$I_{NaK}=(1-af)\cdot I_{NaK}+af\cdot {\bar{I}}_{NaK}.$$

#### 4.4.3. SERCA

The biophysical model of SERCA that we developed by modifying the Tran et al. model [58] was used after adding the β1-adrenergic regulation. SERCA belongs to the same family of the P-type ion pump as the Na^+^/K^+^ ATPase; namely, the activity of SERCA was inhibited by the non-phosphorylated phospholamban (PLB), and SERCA was relieved from this inhibition through phosphorylation of PLB during the β1-AR stimulation. The regulation by AR was defined by decreasing the apparent *K_d_* for the cytosolic Ca^2+^ to half the control (*sf*_SERCA_ = 0.5) when the PLB was phosphorylated. The thermodynamic constraint was satisfied by applying the same *sf* to the rate *k_-1_*, which was used as an adjustable parameter in the original model. The time course of activation was defined by Equation (12) and the amplitude of the Ca^2+^ flux into SR was calculated by Equation (17). The same time constant τ = 1 s was used for the β1-AR regulation of Na^+^/K^+^.

(16) $${\bar{Kd}}_{Cai}=sf_{SERCA}\cdot Kd_{Cqi}.$$

The whole cell *J_SERCA_* was determined as a sum of control *J_SERCA_c_* and activated *J_SERCA_a_*.

(17) $$J_{\mathrm{SERCA}}=(1-af)\cdot J_{\mathrm{SERCA}\_c}+af\cdot J_{SERCA\_a}.$$

#### 4.4.4. LCC

The amplitude of *I*_CaL_ increased during the β1-AR stimulation without a marked change in its time course. Thus, the amplitude of *I*_CaL_ is magnified when noradrenaline is applied to the model cell. We assumed that the fraction of non-phosphorylated population of LCC is 56.5% and shows virtually zero conductance in the absence of β-adrenergic stimulation. Thus, the whole cell conductance of *I*_CaL_ is proportional to *af*_(t)_ (Equation (12)).

#### 4.4.5. α1-Adrenergic Signal, [IP_3_]

We failed to find an α1-AR model to incorporate in the PVC model. In this study, the time-dependent activation and desensitization of the receptor was simply represented by changes in the active second messenger [IP_3a_] using a concentration [IP_3_]_(t)_, and an inactivation (*i*) parameter of the α1-AR at time *t*. To obtain [IP_3_ ], equal to the control 0.015 mM after the desensitization, a lower limit of 0.1 was applied to the range of $i_{\infty }$:

(18) $$[IP_{3a}]=i_{\left(t\right)}\cdot {\left[IP_{3}\right]}_{\left(t\right)},$$

(19) $$i_{\left(t+dt\right)}=i_{\infty }-(i_{\infty }-i_{\left(t\right)})\cdot \exp(-\frac{dt}{\tau_{i}})\;\tau_{i}=30000\;(ms^{-1}),\;0.1\leq i_{\infty }<1.$$

The [IP_3_]_(t)_ at time *t* is also given by an exponential function:

(20) $${\left[IP_{3}\right]}_{\left(t+dt\right)}={\left[IP_{3}\right]}_{\max}-({\left[IP_{3}\right]}_{\max}-{\left[IP_{3}\right]}_{\left(t\right)})\cdot \exp(-\frac{dt}{\tau_{i}{}_{p3}})\;\tau_{ip3}=5000\;(ms^{-1}).$$

#### 4.4.6. InsP_3_R

In the cardiac myocytes, the type II homologous isomer is the major component of InsP_3_R [59] and is expressed on the SR membrane near the T-tubules [7]. Thus, in the model, the InsP_3_R was exposed to *jnc*, so that its Ca^2+^ release plays a critical role in determining the bias level for the CICR. The open probability pO_InsP3R_ was calculated using the InsP_3_R-II model [60]. The influence of Ca^2+^ on the pO_InsP3R_ was much less than that of IP_3_ over the range of [Ca^2+^] during the Ca^2+^ transient. The resting level of [IP_3_] was set to 0.015 μM according to Cooling et al. [17] so that *J*_InsP3R_ was virtually zero. The [IP_3_] was increased 10-fold (0.15 μM) during the stimulation of α1-AR. The Ca^2+^ flux via InsP_3_R channel was calculated using Equation (21).

(21) $$J_{InsP3R}=P_{IP3R}\cdot pO_{InsP3R}\cdot ({\left[Ca^{2+}\right]}_{SRrl}-{\left[Ca^{2+}\right]}_{jnc}).$$

#### 4.4.7. Background K^+^ Current, *I*_Kbg_

Doisne et al. observed a slow continuous depolarization of ~+21 mV during a 15 min application of NA to the rat PV tissue, which was larger than the +12 mV depolarization in the atrial tissue [9]. Similar depolarization was observed in response to phenylephrine (a selective agonist for α1-AR) in the rat atrial tissue preparation by Jahnel et al. [61]. In our simulation, the AR-mediated decrease of the background *I*_Kbg_ was set at 20%–30% with a time constant of 120 s to obtain the experimental time course of depolarization.

(22) $$I_{K_{bg}}=sf_{K_{bg}}\cdot G_{K_{bg}}\cdot (V_{m}-E_{K}),$$

(23) $$sf_{K_{bg}(t+dt)}=sf_{K_{bg}(t)}+(sf_{K_{bg}}{}_{\infty }-sf_{K_{bg}(t)})\cdot \frac{dt}{\tau_{Kb}}\;\tau_{Kb}=80000\;(ms^{-1}).$$

### 4.5. Simulation of the Random Events of CICR 

If [Ca^2+^]*_jnc_* fluctuates over a range slightly lower than the threshold of full CICR activation, the membrane potential may vary randomly. However, in the conventional numerical integration of d[Ca^2+^]/dt, the average time course of [Ca^2+^]*_jnc_* only changes smoothly and continuously. In simulation, the random activation of a couplon could be technically evoked by introducing a random function in the present Hinch format of CICR. For practical purposes, the magnitude of [Ca^2+^]*_jnc_* was multiplied two-fold at a probability of 0.0007 when the random function implemented in the VB system provided a value larger than (1 – 0.0007) at every step of numerical integration. This additional fluctuation randomly induced a full-blown CICR shown in Figure 5. Note, this modified [Ca^2+^]*_jnc_* is only used to determine the opening rate of a couplon, but does not interfere with the mass conservation of Ca^2+^ dynamics.

### 4.6. Changes in Membrane Potential and Ion Concentrations

A C_m_ of 115.3 pF was assumed in the PVC model according to the measurements of Okamoto et al. [7]. The AP was triggered by applying a current pulse of −5 pA/pF for 3 ms. Changes in V_m_ as well as the intracellular ion concentrations were calculated.

(24) $$I_{Ca,tot\_jnc}=I_{Ca,CaL\_jnc}+I_{Ca,NCX\_jnc},$$

(25) $$I_{Ca,tot\_iz}=I_{Ca,CaL\_iz}+I_{Ca,bg\_iz}+I_{Ca,NCX\_iz}+I_{Ca,PMCA\_iz},$$

(26) $$I_{Ca,tot\_blk}=I_{Ca,CaL\_blk}+I_{Ca,bg\_blk}+I_{Ca,NCX\_blk}+I_{Ca,PMCA\_blk},$$

(27) $$I_{Ca,tot}=I_{Ca,tot\_jnc}+I_{Ca,tot\_iz}+I_{Ca,tot\_blk},$$

(28) $$\begin{array}{l} I_{Na,tot}=I_{\mathrm{Na}}+I_{\mathrm{Na},\mathrm{CaL}\_\mathrm{jnc}}+I_{\mathrm{Na},\mathrm{CaL}\_\mathrm{iz}}+I_{\mathrm{Na},\mathrm{CaL}\_\mathrm{blk}}+I_{\mathrm{Na},\mathrm{bg}}\\ +I_{\mathrm{Na},\mathrm{NaK}}+I_{\mathrm{Na},\mathrm{NCX}\_\mathrm{jnc}}+I_{\mathrm{Na},\mathrm{NCX}\_\mathrm{iz}}+I_{\mathrm{Na},\mathrm{NCX}\_\mathrm{blk}} \end{array}$$

(29) $$\begin{array}{l} I_{K,tot}=I_{k,K1}+I_{K,\mathrm{Kr}}+I_{K,\mathrm{Kto}}+I_{K,\mathrm{ur}}+I_{K,\mathrm{bg}}+I_{K,\mathrm{Na}}\\ +I_{K,\mathrm{CaL}\_\mathrm{jnc}}+I_{K,\mathrm{CaL}\_\mathrm{iz}}+I_{K,\mathrm{CaL}\_\mathrm{blk}}+I_{K,\mathrm{NaK}} \end{array}$$

The concentration changes of intracellular ions were calculated by the numerical integration of the membrane fluxes across the surface membrane as well as the SR membrane.

(30) $$\frac{dCa_{tot\_jnc}}{dt}=(-\frac{I_{Ca,tot\_jnc}\cdot C_{m}}{2\cdot F}+J_{Ca\_rel}-J_{Ca\_jnciz}+J_{IP3})/V_{vol\_jnc},$$

(31) $$\frac{dCa_{tot\_iz}}{dt}=(-\frac{I_{Ca,tot\_iz}\cdot C_{m}}{2\cdot F}+J_{Ca\_jnciz}-J_{Ca\_izblk})/V_{vol\_iz},$$

(32) $$\frac{dCa_{tot\_blk}}{dt}=(-\frac{I_{Ca,tot\_blk}\cdot C_{m}}{2\cdot F}-J_{Ca\_SERCA}+J_{Ca\_izblk})/V_{vol\_blk},$$

(33) $$\frac{dCa_{SRup}}{dt}=\frac{J_{Ca\_SERCA}-J_{Ca\_transSR}}{V_{vol\_SRup}},$$

(34) $$\frac{dCa_{tot\_SRrl}}{dt}=\frac{J_{Ca\_transSR}-J_{Ca\_rel}-J_{IP3}}{V_{vol\_SRrl}},$$

(35) $$\frac{dNa}{dt}=\frac{I_{Na,tot\cdot }C_{m}}{F\cdot V_{vol\_cyt}},$$

(36) $$\frac{dK}{dt}=\frac{(I_{K,tot\cdot }+I_{apl})\cdot C_{m}}{F\cdot V_{vol\_cyt}},$$

(37) $$\frac{dV_{m}}{dt}=-(I_{Ca,tot}+I_{Na,tot}+I_{K,tot}+I_{apl}).$$

### 4.7. Bifurcation Analysis on the Ca^2+^ Dynamics Within the Cell in the Absence of Membrane Ionic Fluxes

The model equations were all translated into an ordinary differential equation (ODE) file. Initial values were obtained using a numerical simulation program. Then equilibrium points were calculated using xppaut [62]. When Hopf bifurcation points were obtained, limit cycles were calculated using a conventional computational tool “Auto” to plot a bifurcation diagram. Ca_tot_ (attomole) was composed of various compartments as described by Equation (38).

(38) $$Ca_{tot}={\left[Ca_{tot}\right]}_{jnc}\cdot Vol_{jnc}+{\left[Ca_{tot}\right]}_{iz}\cdot Vol_{iz}+{\left[Ca_{tot}\right]}_{blk}\cdot Vol_{blk}+{\left[Ca_{tot}\right]}_{SRrl}\cdot Vol_{SRrl}+{\left[Ca^{2+}\right]}_{SRup}\cdot Vol_{up}\;(\mathrm{attomole}).$$

In the bifurcation diagram and the main text, the unit of Ca_tot_ is converted to femtomole from attomole.

## Figures and Tables

**Table 1:** Distribution of plasma membrane channels and transporters.

Channels and Transporters	*jnc*	*iz*	*blk*
Ca^2+^ insensitive channels (*I*_Na_, *I*_Kr_, *I*_K1_, *I*_Kto_, *I*_Kur_, *I*_Clh_, *I*_Kbg_, *I*_Nabg_)	-	0.1	0.9
*I*_PMCA_	-	0.1	0.9
*I*_NaK_	-	0.1	0.9
*I*_CaL(LCC)_	0.75	0.15	0.1
*I*_NCX_	0.03	0.25	0.72

## Supplementary Materials

Supplementary materials can be found at https://www.mdpi.com/1422-0067/20/12/2913/s1. Reference [63] appear in the Supplementary Materials.

## Abbreviations

**Abbreviation**	**Definition**
*nd*	Nano-domain
*jnc*	Junction space
*iz*	Intermediate zone
*blk*	Bulk space
*SR_up_*	Ca^2+^ uptake site of sarcoplasmic reticulum
*SR_rl_*	Ca^2+^ releasing site of sarcoplasmic reticulum
CaRU	Ca^2+^ releasing unit
couplon	A cluster of RyRs on SR membrane
Ca_tot_	Total Ca^2+^ content (femtomole)
V_m_	Membrane potential (mV)
C_m_	Membrane capacitance (pF)
TD	Transient membrane depolarization induced by spontaneous Ca^2+^ release through NCX activation
AP_TD_	Action potential triggered by TD
CICR	Ca^2+^-induced Ca^2+^ release
NA	Noradrenaline
ISO	Isoprenaline

Ion channels and transporters in our PVC model

**Abbreviation**	**Definition**	**Reference**
*I*_Na_	Transient component of sodium current	HuVEC model [16]
*I*_CaL (LCC)_	L-type calcium current	HuVEC model [16]
*I*_Kr_	Delayed rectifier potassium current, fast component	HuVEC model [47]
*I*_K1_	Inward rectifier potassium current	HuVEC model [16]
*I*_Kto_	Transient outward potassium current	Pandit et al. [51]
*I*_Kur_	Ultra-rapid outward potassium current	Bondarenko et al. [52]
*I*_Clh_	Hyperpolarization activated chloride current	Experimental data by Okamoto et al. [8]
*I*_Cab_, *I*_Nab_, *I*_Kb_	Background currents for Ca^2+^, Na^+^ and K^+^	Pandit et al. [51]
*I*_NCX_	Na^+^/Ca^2+^ exchange current	HuVEC model [16]
*I*_NaK_	Na^+^/K^+^ pump current	HuVEC model [16]
SERCA	Sarcoplasmic/endoplasmic reticulum calcium pump	HuVEC model [16]
InsP_3_R	Inositol (1,4,5)-trisphosphate receptor	Sneyd et al. [60]
RyR	Ryanodine receptor	HuVEC model [16]

## References

[1] Haissaguerre M., Jais P., Shah D.C., Takahashi A., Hocini M., Quiniou G., Garrigue S., Le Mouroux A., Le Metayer P., Clementy J. (1998). Spontaneous initiation of atrial fibrillation by ectopic beats originating in the pulmonary veins. N. Engl. J. Med..

[2] Maupoil V., Bronquard C., Freslon J.L., Cosnay P., Findlay I. (2007). Ectopic activity in the rat pulmonary vein can arise from simultaneous activation of α—and β1—adrenoceptors. Br. J. Pharm..

[3] Ehrlich J.R., Cha T.J., Zhang L., Chartier D., Melnyk P., Hohnloser S.H., Nattel S. (2003). Cellular electrophysiology of canine pulmonary vein cardiomyocytes: Action potential and ionic current properties. J. Physiol..

[4] Namekata I., Tsuneoka Y., Tanaka H. (2013). Electrophysiological and pharmacological properties of the pulmonary vein myocardium. Biol. Pharm. Bull..

[5] Takahara A., Hagiwara M., Namekata I., Tanaka H. (2014). Pulmonary Vein Myocardium as a Possible Pharmacological Target for the Treatment of Atrial Fibrillation. J. Pharm. Sci..

[6] Malecot C.O., Bredeloux P., Findlay I., Maupoil V. (2015). A TTX-sensitive resting Na^+^ permeability contributes to the catecholaminergic automatic activity in rat pulmonary vein. J. Cardiovasc. Electrophysiol..

[7] Okamoto Y., Takano M., Ohba T., Ono K. (2012). Arrhythmogenic coupling between the Na^+^-Ca^2+^ exchanger and inositol 1,4,5-triphosphate receptor in rat pulmonary vein cardiomyocytes. J. Mol. Cell Cardiol..

[8] Okamoto Y., Kawamura K., Nakamura Y., Ono K. (2014). Pathological impact of hyperpolarization-activated chloride current peculiar to rat pulmonary vein cardiomyocytes. J. Mol. Cell. Cardiol..

[9] Doisne N., Maupoil V., Cosnay P., Findlay I. (2009). Catecholaminergic automatic activity in the rat pulmonary vein: Electrophysiological differences between cardiac muscle in the left atrium and pulmonary vein. Am. J. Physiol. Heart. Circ. Physiol..

[10] Lipp P., Laine M., Tovey S.C., Burrell K.M., Berridge M.J., Li W., Bootman M.D. (2000). Functional InsP3 receptors that may modulate excitation-contraction coupling in the heart. Curr. Biol..

[11] Mackenzie L., Bootman M.D., Laine M., Berridge M.J., Thuring J., Holmes A., Li W.H., Lipp P. (2002). The role of inositol 1,4,5-trisphosphate receptors in Ca^2+^ signalling and the generation of arrhythmias in rat atrial myocytes. J. Physiol..

[12] Jones G., Spencer B.D., Adeniran I., Zhang H. (2012). Development of biophysically detailed electrophysiological models for pacemaking and non-pacemaking human pulmonary vein cardiomyocytes. Conf Proc. IEEE Eng. Med. Biol. Soc..

[13] Seol C.A., Kim J., Kim W.T., Ha J.M., Choe H., Jang Y.J., Shim E.B., Youm J.B., Earm Y.E., Leem C.H. (2008). Simulation of spontaneous action potentials of cardiomyocytes in pulmonary veins of rabbits. Prog. Biophys. Mol. Biol..

[14] Hinch R. (2004). A mathematical analysis of the generation and termination of calcium sparks. Biophys. J..

[15] Hinch R., Greenstein J.L., Tanskanen A.J., Xu L., Winslow R.L. (2004). A simplified local control model of calcium-induced calcium release in cardiac ventricular myocytes. Biophys. J..

[16] Himeno Y., Asakura K., Cha C.Y., Memida H., Powell T., Amano A., Noma A. (2015). A human ventricular myocyte model with a refined representation of excitation-contraction coupling. Biophys. J..

[17] Cooling M., Hunter P., Crampin E.J. (2007). Modeling hypertrophic IP3 transients in the cardiac myocyte. Biophys. J..

[18] Saucerman J.J., Brunton L.L., Michailova A.P., McCulloch A.D. (2003). Modeling beta-adrenergic control of cardiac myocyte contractility in silico. J. Biol. Chem..

[19] Ferrier G.R. (1976). The effects of tension on acetylstrophanthidin-induced transient depolarizations and aftercontractions in canine myocardial and Purkinje tissues. Circ. Res..

[20] Aronson R.S., Gelles J.M. (1977). The effect of ouabain, dinitrophenol, and lithium on the pacemaker current in sheep cardiac Purkinje fibers. Circ. Res..

[21] Kass R.S., Tsien R.W., Weingart R. (1978). Ionic basis of transient inward current induced by strophanthidin in cardiac Purkinje fibres. J. Physiol..

[22] Matsuda H., Noma A., Kurachi Y., Irisawa H. (1982). Transient depolarization and spontaneous voltage fluctuations in isolated single cells from guinea pig ventricles. Calcium-mediated membrane potential fluctuations. Circ. Res..

[23] Ter Keurs H.E., Boyden P.A. (2007). Calcium and arrhythmogenesis. Physiol. Rev..

[24] Cha C.Y., Santos E., Amano A., Shimayoshi T., Noma A. (2011). Time-dependent changes in membrane excitability during glucose-induced bursting activity in pancreatic beta cells. J. Gen. Physiol..

[25] Takeda Y., Shimayoshi T., Holz G.G., Noma A. (2016). Modeling analysis of inositol 1,4,5-trisphosphate receptor-mediated Ca^2+^ mobilization under the control of glucagon-like peptide-1 in mouse pancreatic beta-cells. Am. J. Physiol. Cell. Physiol..

[26] Kurata Y., Tsumoto K., Hayashi K., Hisatome I., Tanida M., Kuda Y., Shibamoto T. (2017). Dynamical mechanisms of phase-2 early afterdepolarizations in human ventricular myocytes: Insights from bifurcation analyses of two mathematical models. Am. J. Physiol. Heart Circ. Physiol..

[27] Shimoni Y., Severson D., Giles W. (1995). Thyroid status and diabetes modulate regional differences in potassium currents in rat ventricle. J. Physiol..

[28] Shinagawa Y., Satoh H., Noma A. (2000). The sustained inward current and inward rectifier K+ current in pacemaker cells dissociated from rat sinoatrial node. J. Physiol..

[29] Severs N.J., Slade A.M., Powell T., Twist V.W., Warren R.L. (1982). Correlation of ultrastructure and function in calcium-tolerant myocytes isolated from the adult rat heart. J. Ultrastruct. Res..

[30] Volders P.G., Vos M.A., Szabo B., Sipido K.R., de Groot S.H., Gorgels A.P., Wellens H.J., Lazzara R. (2000). Progress in the understanding of cardiac early afterdepolarizations and torsades de pointes: Time to revise current concepts. Cardiovasc. Res..

[31] Alfonzo-Mendez M.A., Carmona-Rosas G., Hernandez-Espinosa D.A., Romero-Avila M.T., Garcia-Sainz J.A. (2018). Different phosphorylation patterns regulate alpha1D-adrenoceptor signaling and desensitization. Biochim. Biophys. Acta. Mol. Cell. Res..

[32] Garcia-Sainz J.A., Vazquez-Prado J., del Carmen Medina L. (2000). Alpha 1-adrenoceptors: Function and phosphorylation. Eur. J. Pharm..

[33] Rajagopal S., Shenoy S.K. (2018). GPCR desensitization: Acute and prolonged phases. Cell. Signal.

[34] Abdellatif M.M., Neubauer C.F., Lederer W.J., Rogers T.B. (1991). Angiotensin-induced desensitization of the phosphoinositide pathway in cardiac cells occurs at the level of the receptor. Circ. Res..

[35] Jiang T., Pak E., Zhang H.L., Kline R.P., Steinberg S.F. (1996). Endothelin-dependent actions in cultured AT-1 cardiac myocytes. The role of the epsilon isoform of protein kinase C. Circ. Res..

[36] Zhang S., Hiraoka M., Hirano Y. (1998). Effects of alpha1-adrenergic stimulation on L-type Ca2+ current in rat ventricular myocytes. J. Mol. Cell. Cardiol..

[37] Terzic A., Puceat M., Clement O., Scamps F., Vassort G. (1992). Alpha 1-adrenergic effects on intracellular pH and calcium and on myofilaments in single rat cardiac cells. J. Physiol..

[38] Miki K., Yoshimoto M. (2013). Sympathetic nerve activity during sleep, exercise, and mental stress. Auton. Neurosci..

[39] Hartmann H.A., Mazzocca N.J., Kleiman R.B., Houser S.R. (1988). Effects of phenylephrine on calcium current and contractility of feline ventricular myocytes. Am. J. Physiol..

[40] Hescheler J., Nawrath H., Tang M., Trautwein W. (1988). Adrenoceptor-mediated changes of excitation and contraction in ventricular heart muscle from guinea-pigs and rabbits. J. Physiol..

[41] Ertl R., Jahnel U., Nawrath H., Carmeliet E., Vereecke J. (1991). Differential electrophysiologic and inotropic effects of phenylephrine in atrial and ventricular heart muscle preparations from rats. Naunyn. Schmiedebergs Arch. Pharm..

[42] Jahnel U., Duwe E., Pfennigsdorf S., Nawrath H. (1994). On the mechanism of action of phenylephrine in rat atrial heart muscle. Naunyn Schmiedebergs Arch. Pharm..

[43] Schumann H.J., Wagner J., Knorr A., Reidemeister J.C., Sadony V., Schramm G. (1978). Demonstration in human atrial preparations of alpha-adrenoceptors mediating positive inotropic effects. Naunyn Schmiedebergs Arch. Pharm..

[44] Skomedal T., Aass H., Osnes J.B., Fjeld N.B., Klingen G., Langslet A., Semb G. (1985). Demonstration of an alpha adrenoceptor-mediated inotropic effect of norepinephrine in human atria. J. Pharm. Exp..

[45] Wang Y.G., Dedkova E.N., Ji X., Blatter L.A., Lipsius S.L. (2005). Phenylephrine acts via IP3-dependent intracellular NO release to stimulate L-type Ca2+ current in cat atrial myocytes. J. Physiol.

[46] Jahnel U., Jakob H., Nawrath H. (1992). Electrophysiologic and inotropic effects of alpha-adrenoceptor stimulation in human isolated atrial heart muscle. Naunyn Schmiedebergs Arch. Pharm..

[47] Asakura K., Cha C.Y., Yamaoka H., Horikawa Y., Memida H., Powell T., Amano A., Noma A. (2014). EAD and DAD mechanisms analyzed by developing a new human ventricular cell model. Prog. Biophys. Mol. Biol..

[48] Song Y., Hao G., Boyett M., Yang X., Du Y., Shui Z. (2009). Action potential, sodium and gap junction channels in rat pulmonary vein myocytes. Proc. Physiol. Soc..

[49] Boyle W.A., Nerbonne J.M. (1992). Two functionally distinct 4-aminopyridine-sensitive outward K+ currents in rat atrial myocytes. J. Gen. Physiol..

[50] Pond A.L., Scheve B.K., Benedict A.T., Petrecca K., Van Wagoner D.R., Shrier A., Nerbonne J.M. (2000). Expression of distinct ERG proteins in rat, mouse, and human heart. Relation to functional I_Kr_ channels. J. Biol. Chem..

[51] Pandit S.V., Clark R.B., Giles W.R., Demir S.S. (2001). A mathematical model of action potential heterogeneity in adult rat left ventricular myocytes. Biophys. J..

[52] Bondarenko V.E., Szigeti G.P., Bett G.C., Kim S.J., Rasmusson R.L. (2004). Computer model of action potential of mouse ventricular myocytes. Am. J. Physiol. Heart Circ. Physiol..

[53] Kuzumoto M., Takeuchi A., Nakai H., Oka C., Noma A., Matsuoka S. (2008). Simulation analysis of intracellular Na^+^ and Cl^−^ homeostasis during β1-adrenergic stimulation of cardiac myocyte. Prog. Biophys. Mol. Biol..

[54] Himeno Y., Sarai N., Matsuoka S., Noma A. (2008). Ionic mechanisms underlying the positive chronotropy induced by beta1-adrenergic stimulation in guinea pig sinoatrial node cells: A simulation study. J. Physiol. Sci..

[55] Oka C., Cha C.Y., Noma A. (2010). Characterization of the cardiac Na+/K+ pump by development of a comprehensive and mechanistic model. J. Biol..

[56] Smith N.P., Crampin E.J. (2004). Development of models of active ion transport for whole-cell modelling: Cardiac sodium-potassium pump as a case study. Prog. Biophys. Mol. Biol..

[57] Despa S., Bossuyt J., Han F., Ginsburg K.S., Jia L.G., Kutchai H., Tucker A.L., Bers D.M. (2005). Phospholemman-phosphorylation mediates the beta-adrenergic effects on Na/K pump function in cardiac myocytes. Circ. Res..

[58] Tran K., Smith N.P., Loiselle D.S., Crampin E.J. (2009). A thermodynamic model of the cardiac sarcoplasmic/endoplasmic Ca^2+^ (SERCA) pump. Biophys. J..

[59] Miyakawa T., Maeda A., Yamazawa T., Hirose K., Kurosaki T., Iino M. (1999). Encoding of Ca^2+^ signals by differential expression of IP3 receptor subtypes. EMBO J..

[60] Sneyd J., Dufour J.F. (2002). A dynamic model of the type-2 inositol trisphosphate receptor. Proc. Natl. Acad. Sci. USA.

[61] Jahnel U., Nawrath H., Carmeliet E., Vereecke J. (1991). Depolarization-induced influx of sodium in response to phenylephrine in rat atrial heart muscle. J. Physiol..

[62] Ermentrout B. (2002). Simulating, Analyzing, and Animating Dynamical Systems: A Guide to XPPAUT for Researchers and Students. Software. Environments and Tools.

[63] Yan D.H., Ishihara K. (2005). Two Kir2.1 channel populations with different sensitivities to Mg^2+^ and polyamine block: A model for the cardiac strong inward rectifier K^+^ channel. J. Physiol..

