# Supplementary material for: Mechanisms Underlying Spontaneous Action Potential Generation Induced by Catecholamine in Pulmonary Vein Cardiomyocytes: A Simulation Study
Source: Int J Mol Sci. 2019 Jun 14;20(12):2913. doi: 10.3390/ijms20122913 (PMC6628582; doi:10.3390/ijms20122913)
Supplement: Supplementary file 1 [file ijms-20-02913-s001.pdf]

Supporting Materials

The source code of the computer model can be downloaded from our “e-Heart” website (http://www.eheartsim.com).

1. Abbreviations

Table S1. Abbreviations in model equations

V_m	membrane potential (mV)
I_{tot_cell}	total current of ion channels and exchangers (pA/pF)
$I_{tot\_X\_a}$	total current of ion ‘X’ channels and exchangers at space ‘a’ (pA/pF)
I_{app_blk}	current applied through a patch electrode (pA/pF)
E_X	reversal potential of ion ‘X’ , determined by the Nernst equation (mV)
C_m	Whole cell membrane capacitance (pF)
G_I	conductance of current ‘I’ (nS/mV)
GHK_{X_a}	a modified Goldman-Hodgkin-Katz equation of ion ‘X’ at space ‘a’ (mM)
k, α, β, ν	rate constants (/ms)
K_{d_X}	dissociation constant for ion ‘X’ (mM)
$P_{I(\_X)}$	converting factor of current ‘I’ from GHK_X (pA/pF/mM)
ν_{cyc_T}	turnover rate of transporter ‘T’ (/ms)
$p(S)_{(\_a)}$	probability of state ‘S’ in a scheme of state transitions at space ‘a’
V_X	total volume of space ‘X’ (picoL)
$[X_{total}]_a$	total concentration of substance ‘X’ at space ‘a’ (mM)
$[X_{free}]_a$	free concentration of substance ‘X’ at space ‘a’ (mM)
$[X]_a$	concentration of ‘X’ at space ‘a’ (mM)
J_X	total flux of ion ‘X’ (attomol/ms)
z_X	valence of ion ‘X’
$\frac{d[X]_a}{dt}$	rate of change of ‘X’ concentration at space ‘a’ (mM/ms)

2. Model parameters

Physical constants

Table S2 Physical constants

R	8.3143	C·mV/mmol/K
T	310	K
F	96.4867	C/mmol

Ion concentrations

Table S3 Ionic composition of external solution

$[K^+]_o$	5.4	mM
$[Na^+]_o$	140	mM
$[Ca^{2+}]_o$	1.8	mM

Table S4. Cell Volume, volumes of Ca^{2+} compartments and cell capacitance

V_{cell} (pL)	V_{jnc}	V_{iz}	V_{blk}	V_{SR}	V_{SRrl}	V_{SRup}	C_m (pF)
22373	$0.008 \cdot V_{cell}$	$0.035 \cdot V_{cell}$	$0.68 \cdot V_{cell}$	$0.072 \cdot V_{cell}$	$0.225 \cdot V_{SR}$	$0.775 \cdot V_{SR}$	113.5514

As the capacitance of PVC model is 113.55, assuming Sc_cell = 0.59 compared to a standard V_{cell} (V_{std} = 37.92 pL) and a C_m (C_{std} = 192.46 pF) of a human ventricular cell, all Ca^{2+} fluxes between Ca^{2+} compartments and the cell volume are scaled by using Sc_cell.

3. Ca^{2+}-binding proteins and diffusion

The same species and concentrations of buffering proteins, and diffusion constants between compartments (between jnc-iz, iz-blk, and SRup-SRrl) are assumed as in HuVEC model [1].

4. GHK equation and Nernst equation

The same equations in HuVEC model [1] are used for calculating GHK and Nernst equations.

5. Ion channels and transporters

I_{Na}

I_{NaT} model in HuVEC model [1] is used. The rates k_{OI2}, k_{Isb} and k_{Isf} are multiplied by 0.1, 2 and 2, respectively and the voltage dependency of the reaction rate between O and I_2 states, and transition rate of the I_s state are shifted negatively by 15 mV, respectively to fit duration of the action potential and activation of rat PVC. P_{Na} is slightly increased to 9.4584.

I_{CaL}

I_{CaL} model in HuVEC model [1] is used.

$$I_{CaL\_Ca} = P_{CaL} \cdot pO \cdot GHKCa\,,\ P_{CaL} = 9.4780 \qquad S(1)$$

The gating of I_{CaL} model was largely simplified to adapt to the Hinch model (2004) [13-14] of CaRU in the HuVEC model. The gating was determined by a V_m-dependent gate and a Ca-dependent gate. The V_m-dependent gate was described by assuming two state transition scheme. Both of opening and closing rates are purely V_m-dependent (a_+ and a_-).

Voltage-gate

$$\alpha_{+} = \frac{1}{3.734 \cdot \exp(-\frac{V_m}{8.5} + 0.35 \cdot \exp(-\frac{V_m}{3500})} \qquad \text{S(2)}$$

$$\alpha_{-} = \frac{1}{4.65 \cdot \exp(\frac{V_m}{15}) + 1.363 \cdot \exp(\frac{V_m}{100})} \qquad \text{S(3)}$$

Ca^{2+}-gate

It was assumed that the Ca^{2+}-binding site of LCC for channel closing might be located very close to the exit of the channel pore, and the local Ca^{2+} concentration in a hypothetical nano-domain was assumed ($[Ca^{2+}]_{nd}$) in the CaRU model. Under the assumption of a minimum volume of nano-domain, $[Ca^{2+}]_{nd}$ was successfully approximated by the equation,

$$[Ca]_{nd} = \frac{[Ca]_{df} + \frac{J_R}{g_D} \cdot [Ca]_{SRrel} + \frac{J_L}{g_D} \frac{\delta V \cdot e^{-\delta V}}{1 - e^{-\delta V}} [Ca]_o}{1 + \frac{J_R}{g_D} + \frac{J_L}{g_D} \frac{\delta V}{1 - e^{-\delta V}}} \qquad \text{S(4)}$$

In this equation, $[Ca^{2+}]_{nd}$ is determined as a weighted average of $[Ca^{2+}]$ in the three spaces ($[Ca^{2+}]_{df}$, $[Ca^{2+}]_{SRrl}$), which are directly connected to the nano-domain. $[Ca^{2+}]_{ds}$ is either the $[Ca^{2+}]_{jnc}$ in the case of dyadic I_{CaL}, or $[Ca^{2+}]_{iz}$ and $[Ca^{2+}]_{blk}$ in the case of I_{CaL} located on the cell surface membrane of the *iz* and *blk* spaces, respectively. $[Ca^{2+}]_{SRrel}$ is connected through the conductance J_R of the couplon consisting of a cluster of RyRs, and $[Ca^{2+}]_o$ through the conductance (J_L) of I_{CaL}.
Since LCC is not coupled to RyRs outside of dyadic junction, $[Ca^{2+}]_{nd}$ is determined by the following equation S(5).

$$[Ca]_{nd} = \frac{[Ca]_{df} + \frac{J_L}{g_D} \frac{\delta V \cdot e^{-\delta V}}{1 - e^{-\delta V}} [Ca]_o}{1 + \frac{J_L}{g_D} \frac{\delta V}{1 - e^{-\delta V}}} \qquad \text{S(5)}$$

The Ca^{2+}-gate was also described by a two-state transition scheme with closing rate (k_{oc}) and opening rate (k_{co}). Thus, I_{CaL} gating is described by a four-state transition model. k_{oc} is,

$$k_{oc} = \frac{[Ca^{2+}]_{nd} \cdot \alpha_{+}}{TL \cdot K_L} \quad , \qquad K_L = 0.00154 \quad TL_{-} = 147.51 \qquad \text{S(6)}$$

As appeared in this equation, the opening rate α_{+} of the V-gate is used for k_{oc}, assuming that the

opening of the V_m-gate facilitates the Ca^{2+}-mediated inactivation of I_{CaL}.

It is also assumed that the removal rate of the inactivation (k_{co}) is dependent on Vm, and thereby the 'steady-state inactivation' curve could be simulated.

$$k_{co} = Eta1 + Eta2 \qquad \text{S(7)}$$

$$Eta1 = \frac{1}{8084 \cdot \exp(\frac{V_m}{10}) + 158 \cdot \exp(\frac{V_m}{1000})} \qquad \text{S(8)}$$

$$Eta2 = \frac{1}{134736 \cdot \exp(-\frac{V_m}{5}) + 337 \cdot \exp(-\frac{V_m}{2000})} \qquad \text{S(9)}$$

Eta2 was used to reconstruct the non-inactivated component of I_{CaL} on larger depolarizations.

I_{Kr}

I_{Kr} model in HuVEC model [2] is used. The current amplitude is described with an Ohmic equation.

$$I_{Kr} = G_{Kr} \cdot (V_m - E_K) \cdot p(O)_{Kr} \cdot \left(\frac{[K^+]_o}{4.5}\right)^{0.2}, \qquad G_{Kr} = 0.27356 \qquad \text{S(10)}$$

The open probability of the channel is described with three gating parameters, y_1, y_2 and y_3.

$$p(O)_{Kr} = (0.6 \cdot y_1 + 0.4 \cdot y_2) \cdot y_3 \qquad \text{S(11)}$$

$$\frac{dy_1}{dt} = \alpha_{y1} \cdot (1.0 - y_1) - \beta_{y1} \cdot y_1 \qquad \text{S(12)}$$

$$\frac{dy_2}{dt} = \alpha_{y2} \cdot (1.0 - y_2) - \beta_{y2} \cdot y_2 \qquad \text{S(13)}$$

$$\frac{dy_3}{dt} = \alpha_{y3} \cdot (1.0 - y_3) - \beta_{y3} \cdot y_3 \qquad \text{S(14)}$$

$$\alpha_{y1} = \frac{1}{35 \cdot \exp\left(-\frac{V_m}{10.5}\right) + 75 \cdot \exp\left(-\frac{V_m}{100}\right)} \qquad \beta_{y1} = \frac{1}{470 \cdot \exp\left(\frac{V_m}{8.3}\right) + 220 \cdot \exp\left(\frac{V_m}{29}\right)} \qquad \text{S(15)}$$

$$\alpha_{y2} = \frac{1}{350 \cdot \exp\left(-\frac{V_m}{10.5}\right) + 300 \cdot \exp\left(-\frac{V_m}{100}\right)} \qquad \beta_{y2} = \frac{1}{1850 \cdot \exp\left(\frac{V_m}{8.3}\right) + 2200 \cdot \exp\left(\frac{V_m}{29}\right)} \qquad \text{S(16)}$$

$$\alpha_{y3} = \frac{1}{0.015 \cdot \exp\left(\frac{V_m}{6}\right) + 7 \cdot \exp\left(\frac{V_m}{60}\right)} \qquad \beta_{y3} = \frac{1}{0.114 \cdot \exp\left(-\frac{V_m}{9.2}\right) + 2.3 \cdot \exp\left(-\frac{V_m}{1000}\right)} \qquad \text{S(17)}$$

I_{K1}

The I_{K1} model developed by Yan and Ishihara (2005) [61] was used in HuVEC model (1) after modifying several parameters.

$$I_{K1\_K\_cyt} = G_{K1} \cdot (V_m - E_K) \cdot p(O)_{K1} \quad S(18)$$

$$G_{K1} = \frac{0.03182 \cdot \left(\frac{[K^+]_o}{4.5} \right)^{0.4}}{1.0 + \exp\left(-\frac{[K^+]_o - 2.2}{0.6} \right)} \quad S(19)$$

$$p(O)_{K1} = po_{mode1} + po_{mode2} \quad S(20)$$

$$f_{mode1} = 0.9 \quad S(21)$$

$$po_{mode1} = f_{mode1} \cdot (1 - Pb_{spm}) \cdot \left(po_{Mg} + \frac{2}{3} \cdot po_{Mg1} + \frac{1}{3} \cdot po_{Mg2} \right) \quad S(22)$$

$$po_{mode2} = \frac{(1 - f_{mode1})}{1.0 + \frac{[SPM]}{40.0 \cdot \exp\left(-\frac{V_m - E_K}{9.1} \right)}}, \; [SPM] = 5 \quad (\mu M) \quad S(23)$$

The Mg^{2+}-block in the mode1

$$po_{Mg} = f_O \cdot f_O \cdot f_O \quad S(24)$$

$$po_{Mg1} = 3.0 \cdot f_O \cdot f_O \cdot f_B \quad S(25)$$

$$po_{Mg2} = 3.0 \cdot f_O \cdot f_B \cdot f_B \quad S(26)$$

$$f_O = \frac{\alpha_{Mg}}{\alpha_{Mg} + \beta_{Mg}}, \quad f_B = \frac{\beta_{Mg}}{\alpha_{Mg} + \beta_{Mg}} \quad S(27)$$

$$\alpha_{Mg} = 12.0 \cdot \exp(-0.025 \cdot (V_m - E_K)) \quad S(28)$$

$$\beta_{Mg} = 28 \cdot [Mg^{2+}]_{cyt} \cdot \exp(0.025 \cdot (V_m - E_K)) \quad S(29)$$

The SPM-block in the mode1

$$\frac{dPb_{spm}}{dt} = \beta_{SPM} \cdot po_{Mg} \cdot (1 - Pb_{spm}) - \alpha_{SPM} \cdot Pb_{spm} \quad S(30)$$

$$\alpha_{SPM} = \frac{0.17 \cdot \exp\left(-0.07 \cdot \left((V_m - E_K) + 8 \cdot [Mg^{2+}]_{cyt}\right)\right)}{1.0 + 0.01 \cdot \exp\left(0.12 \cdot \left((V_m - E_K) + 8 \cdot [Mg^{2+}]_{cyt}\right)\right)} \quad S(31)$$

$$\beta_{SPM} = \frac{0.28 \cdot [SPM] \cdot \exp\left(0.15 \cdot \left((V_m - E_K) + 8 \cdot [Mg^{2+}]_{cyt}\right)\right)}{1.0 + 0.01 \cdot \exp\left(0.13\left((V_m - E_K) + 8 \cdot [Mg^{2+}]_{cyt}\right)\right)} \quad S(32)$$

I_{Kto}

I_{Kto} model in a rat ventricular cell model [3] (Pandit et al.) is used after a few modifications. G_{IKto} is increased from 0.035 to 0.135226 (μS = pA/mV). The fast s gate is removed and τ of slow s gate is multiplied by 10.

$$I_{Kto} = G_{Kto} \cdot p_r \cdot s_s) \cdot (V_m - E_K) \quad S(33)$$

$$p_{r\infty} = \frac{1}{1 + Exp(-\frac{V_m + 10.6}{11.42})} \quad S(34)$$

$$\tau_{pr} = \frac{1000}{45.16 \cdot Exp(0.03577 \cdot (V_m + 50)) + 98.9 \cdot Exp(-0.1 \cdot (V_m + 38))} \quad S(35)$$

$$s_{s\infty} = \frac{1}{1 + Exp(\frac{V_m + 45.3}{6.8841})} \qquad \tau_{ss} = 10000 \cdot 3.7 \cdot Exp(-(\frac{V_m + 70}{30})^{\wedge 2} + 0.035) \quad S(36)$$

$$\frac{dp_r}{dt} = \frac{p_{r\infty} - p_r}{\tau_r} \qquad \frac{ds_s}{dt} = \frac{s_{s\infty} - s_s}{\tau_s} \quad S(37)$$

I_{Kur}

I_{Kur} model in a mouse ventricular cell model [4] (Bondarenko et al.) is used after reducing G_{Kur} to 0.42 % (0.0672) of its original value.

$$I_{Kur} = G_{Kur} \cdot a_{ur} \cdot i_{ur} \cdot (V_m - E_K) \quad S(38)$$

$$a_{ur\infty} = \frac{1}{1 + Exp(-\frac{V_m + 22.5}{7.7})} \qquad \tau_{a,ur} = 0.493 \cdot Exp(-0.0692 \cdot V_m) + 2.058 \quad S(39)$$

$$i_{ur\infty} = \frac{1}{1 + Exp(\frac{V_m + 45.2}{5.7})} \qquad \tau_{i,ur} = 1200 - \frac{170}{1 + Exp(\frac{V_m + 45.2}{5.7})} \quad S(40)$$

$$\frac{da_{urt}}{dt} = \frac{a_{ur\infty} - a_{ur}}{\tau_{a,ur}} \qquad \frac{di_{urt}}{dt} = \frac{i_{ur\infty} - i_{ur}}{\tau_{i,ur}} \quad S(41)$$

I_{Clh}

I_{Clh} model is newly developed based on experimental report by Okamoto et al. [5]. The model is a 3-state model as Fig. S1A. The current traces calculated by the I_{Clh} model using E_{Cl} = -20 mV is shown in Fig. S1B. I_{Clh} is not included in determining the membrane potential in the model. If the I_{Clh} was included, the membrane potential was slightly depolarized by a few mV. The automaticity and the action potential configuration did not obviously change because the activation range of I_{Clh} was much more negative compared to the V_m range of our PVC model.

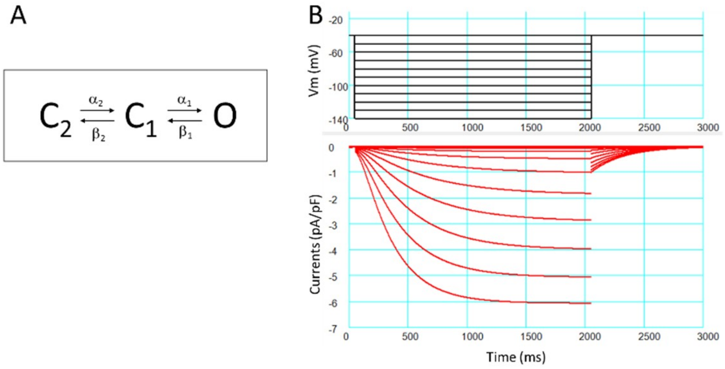

Fig. S1 Model scheme and current traces of I_{Clh}.

$$I_{Clh} = G_{Clh} \cdot O \cdot \left(V_m - E_{Cl}\right),\ E_{Cl} = -20\ \text{(mV)} \qquad \text{S(42)}$$

$$O = 1 - C_1 - C_2 \qquad \text{S(43)}$$

$$\frac{dC_2}{dt} = -\alpha_2 \cdot C_2 + \beta_2 \cdot C_1 \qquad \text{S(44)}$$

$$\frac{dC_1}{dt} = \alpha_2 \cdot C_2 - (\alpha_1 + \beta_2) \cdot C_1 + \beta_1 \cdot O \qquad \text{S(45)}$$

$$\alpha_1 = 0.000064 \cdot e^{-0.028 \cdot V_m} \qquad \text{S(46)}$$

$$\alpha_2 = 0.0008 \cdot e^{-0.02 \cdot V_m} \qquad \text{S(47)}$$

$$\beta_1 = 0.0075 \cdot e^{0.018 \cdot V_m} \qquad \text{S(48)}$$

$$\beta_2 = \frac{0.001}{0.0006 \cdot e^{-\frac{V_m}{15.6}} + 0.0594 \cdot e^{-\frac{V_m}{126}}} \qquad \text{S(49)}$$

Background currents (Ca^{2+}, Na^{+} and K^{+})

Background currents of ion ‘X’ {X = Ca^{2+}, Na^{+} and K^{+}} were calculated by the conventional format with a scaling format ($scf_{X,bg}$).

$$I_{X,bg} = G_{X,bg} \cdot scf_{X,bg} \cdot (V_m - E_K) \qquad X = \{Na, K, Ca\} \qquad \text{S(50)}$$

$$G_{Cabg} = 1.4028 \cdot 10^{-4}, \quad G_{Nabg} = 3.7206 \cdot 10^{-3}, \quad G_{Kbg} = 2.5883 \cdot 10^{-2}, \quad (pA/pF/mV)$$

The $scf_{Ca,bg}$ was varied to modify the total Ca content within the cell.

To examine the effect of depressing $I_{K,bg}$ during the activation of the α-NA stimulation, the $scf_{K,bg}$ was decreased in several simulation.

$$scf_{K,bg(t)} = scf_{K,bg(t)} + (scfI_{Kb\_inf} - scf_{K,bg(t)}) \cdot \frac{dt}{\tau_{Kb}} \qquad \text{S(51)}$$

A τ_{Kb} of 8000 (/ms) was used.

I_{NCX}, I_{NaK}, PMCA and SERCA

Models for I_{NCX}, I_{NaK} and SERCA are taken from HuVEC [1] after changing the maximum conductances to 36, 144.5 and 140 %, respectively. Conductance of PMCA is not changed from HuVEC model [1]. Fractions of NCX in *jnc*, *iz* and *blk* are changed to 0.03, 0.25 and 0.72, respectively.

InsP3R

The type2 $InsP_3R$ model in Sneyd et al. [5] is adopted without any change. Five states are assumed in the Sneyd model and the state transition of the receptor is regulated by the cytosolic [Ca^{2+}] in addition to the [IP_3] in the cytosol (for detail kinetics see the original paper). The Ca^{2+} flux via the whole cell InsP3R (J_{IP3R}) was calculated using a maximum whole cell conductance (P_{max} = 80 attomoles /ms) and the open probability of the $InsP_3R$ channel (*pO*). The Ca^{2+} concentrations in *jnc* and SR_{rl} were used to calculate the driving force.

$$J_{ip3R} = P_{\max} \cdot pO \cdot ([Ca^{2+}]_{SRrl} - [Ca^{2+}]_{jnc}) \qquad \text{S(52)}$$

CaRU

RyR and LCC

The same RyR and LCC models are used as in HuVEC model [1].

$[Ca^{2+}]_{nd\_noise}$

For the random activation of couplons, a noisy [Ca^{2+}] $Ca00_{\_noise}$ is assumed by introducing a random function N_{RND} ($0 < N_{RND} < 1$). The Ca00 at a step of numerical integration (dt = 0.2 ms) was multiplied by 2 at a probability of 0.0007 to reproduce the variations in latency of the spontaneous

activity after the application of NA.

$$Ca_{00\_noise} = 2 \cdot Ca_{00} \tag{S(53)}$$

6. β1-Adrenergic signaling network

Table S5. Abbreviations for β1-Adrenergic receptor network

Abbreviations	Definitions
β1-Adrenergic receptor	
[L]	isoproterenol (ligand) (mM)
[L_{tot}]	total isoproterenol (total ligand) (mM)
[R]	free β1-adrenergic receptor (mM)
[R_{tot}]	total β1-adrenergic receptor, 0.0000132 (mM)
[LR]	β1-adrenergic receptor bound with ligand (mM)
[LRG]	β1-adrenergic receptor bound with ligand and Gs (mM)
[RG]	β1-adrenergic receptor bound with Gs (mM)
[G]	α, β and γ subunit of Gs (mM)
[G_{tot}]	total Gs, 0.00383 (mM)
[$G_{\beta\gamma}$]	β and γ subunit of Gs (mM)
[R_{act}]	active β1-adrenergic receptor (mM)
βARK	β1-adrenergic receptor kinase
[R_{S301}]	β1-adrenergic receptor phosphorylated at S301 by PKA (mM)
[R_{S464}]	β1-adrenergic receptor phosphorylated at S464 by βARK (mM)
[$G_{\alpha}GTP$]	GTP-bound α subunit of Gs (mM)
[$G_{\alpha}GTP_{tot}$]	total GTP-bound α subunit of Gs (mM)
[$G_{\alpha}GDP$]	GDP-bound α subunit of Gs (mM)
adenylate cyclase	
[AC]	adenylate cyclase (mM)
[AC_{tot}]	total adenylate cyclase, 0.0000497 (mM)
[$G_{\alpha}GTP\_AC$]	adenylate cyclase-bound with $Gs_{\alpha}GTP$ (mM)
[$cAMP_{tot}$]	total cAMP (mM)
[PDE]	phosphodiesterase, 0.000039 (mM)
protein kinase PKA	
[Cat]	catalytic subunit of protein kinase A (mM)
[PKA_{tot}]	total protein kinase A (dimer), 0.001 (mM)
[Cat_{tot}]	total of catalytic subunit of PKA, 0.002 (mM)
[RC]	regulatory subunit bound with catalytic subunit (mM)
[ARC]	RC bound with a cAMP (mM)
[A_2RC]	RC bound with 2 cAMP (mM)
[A_2R]	regulatory subunitc bound with 2 cAMP (mM)
[Cat_{123}]	catalytic subunit in the state Cat_1, Cat_2 and Cat_3 (mM)
[Cat_{45}]	catalytic subunit in the state Cat4 and Cat5 (mM)
[C_PKI]	C bound with PKI (mM)
[PKI_{tot}]	total protein kinase inhibitor, 0.00018 (mM)

β1-adrenergic receptor, activation and desensitization

$$[R_{tot}] = [R_{act}] + ([R_{S464}] + [R_{S301}]) \quad S(54)$$

$$[R_{act}] = [R] + [LR] + [LRG] + [RG] \quad S(55)$$

$$[G_{tot}] = [G] + [RG] + [LRG] + [G_{\beta\gamma}] \quad S(56)$$

Instantaneous equilibrium equations S(57)-S(59) are assumed for the binding of L, R and G

$$[L] \cdot [R] = Kd_{LR} * [LR], \qquad Kd_{LR} = 0.001 \text{ (mM)} \quad S(57)$$
(modified from $K_L = 0.000285$ in Saucerman model [6])

$$[G] \cdot [LR] = Kd_{GLR} * [LRG], \qquad Kd_{GLR} = 0.000062 \text{ (mM)} \quad S(58)$$
(modified from $K_R = 0.062$ in Kuzumoto model [7])

$$[G] \cdot [R] = Kd_{GR} * [RG], \; KdGR = 0.033 \text{ (mM)} \quad S(59)$$
(as in Saucerman model [6])

$$R + LR + LRG + RG = R_{act} \quad S(60)$$

$$R + \frac{L}{Kd_{LR}} R + \frac{G}{Kd_{GLR}} \cdot \frac{L}{Kd_{LR}} R + \frac{G}{Kd_{GR}} R = R_{act} \quad S(61)$$

$$R = \frac{R_{act}}{1 + \frac{L}{Kd_{LR}} + \frac{G}{Kd_{GLR}} \cdot \frac{L}{Kd_{LR}} + \frac{G}{Kd_{GR}}} \quad S(62)$$

R is determined by Eq. S(62) and LR, LRG and RG are determined by Eqs. S(57)-S(59) when the initial values of G and L are given as G_{tot} and L_{tot}, respectively.

Concentrations of β-ARs phosphorylated at S464 & S301 (Eqs. S(63) and S(64)) and concentrations of G protein subunit (total GTP-bound G_α, $G_{\beta\gamma}$ and GDP-bound G_α, Eqs. S(65) – (67)) are calculated by the numerical equations.

$$d[R_{S464}] / dt = 0.0000011 \cdot ([LR] + [LRG]) - 0.0000022 \cdot [R_{S464}] \quad S(63)$$

$$d[R_{S301}] / dt = 0.0036 \cdot [Cat] \cdot [R_{act}] - 0.0000002232 \cdot [R_{S301}] \quad S(64)$$

$$d[G_\alpha GTP_{tot}] / dt = 0.016 \cdot ([RG] + [LRG]) - 0.001 \cdot [G_\alpha GTP_{tot}] \quad S(65)$$

$$d[G_{\beta\gamma}] / dt = 0.016 \cdot ([RG] + [LRG]) - 1200 \cdot [G_\alpha GDP] \cdot [G_{\beta\gamma}] \quad S(66)$$

$$d[G_\alpha GDP] / dt = 0.001 \cdot [G_\alpha GTP_{tot}] - 1200 \cdot [G_\alpha GDP] \cdot [G_{\beta\gamma}] \quad S(67)$$

Activation of AC and cAMP synthesis

The activation of AC is mediated by the binding of AC with $G_\alpha GTP$.

This binding reaction is calculated by assuming an instantaneous equilibrium (Eq. S(68)) between $G_\alpha GTP$ and AC.

$[G_\alpha GTP\_AC] = [G_\alpha GTP] \cdot [AC] / Kd_{ACG}$, $Kd_{ACG} = 0.315$ S(68)

The mass conservation equations are

$[AC_{tot}] = [AC] + [G_\alpha GTP\_AC]$ S(69)

$[G_\alpha GTP_{tot}] = [G_\alpha GTP] + [G_\alpha GTP\_AC]$ S(70)

Solution of the Eqs. S(68)-S(70)

From Eqs. S(68) and S(69),

$[AC_{tot}] - [G_\alpha GTP_{tot}] = [AC] - [G_\alpha GTP]$ S(71)

From Eqs. S(69) and S(70),

$[G_\alpha GTP_{tot}] = [G_\alpha GTP] + [G_\alpha GTP] \cdot [AC] / Kd_{ACG}$ S(72)

From Eqs. S(71) and S(72),

$[G_\alpha GTP_{tot}] = [G_\alpha GTP] + [G_\alpha GTP] \cdot ([AC_{tot}] - [G_\alpha GTP_{tot}] + [G_\alpha GTP]) / Kd_{ACG}$

$[G_\alpha GTP_{tot}] = [G_\alpha GTP] + [G_\alpha GTP] \cdot ([AC_{tot}] - [G_\alpha GTP_{tot}]) / Kd_{ACG} + [G_\alpha GTP]^2 / Kd_{ACG}$ S(73)

$[G_\alpha GTP]^2 / Kd_{ACG} + (1 + ([AC_{tot}]- [G_\alpha GTP_{tot}]) / Kd_{ACG}) * [G_\alpha GTP] - [G_\alpha GTP_{tot}] = 0$

Eq. S(73) is an equation of the 2nd degree for $G_\alpha GTP$ (X).

$G_\alpha GTP$ = X = (-b + math.sqr (b^2 - 4ac)) / 2a

$a = 1/Kd_{ACG}$,

$b = 1 + ([AC_{tot}] - [G_\alpha GTP_{tot}]) / Kd_{ACG}$, S(74)

$c = - [G_\alpha GTP_{tot}]$

When $G_\alpha GTP$ is obtained, [AC] and $[G_\alpha GTP\_AC]$ are determined by Eqs. (18) and (19).

From Eq. S(71),

$[AC] = [AC_{tot}] - [G_\alpha GTP_{tot}] + [G_\alpha GTP]$ S(75)

From Eq. S(72),

$[G_\alpha GTP\_AC] = [G_\alpha GTP_{tot}] - [G_\alpha GTP]$ S(76)

The evolution of total cAMP concentration is calculated by a kinetic equation S(77).

$d[cAMP_{tot}] / dt = 0.0001307 \cdot [AC] \cdot [ATP_{tot}]_i / (1.03 + [ATP_{tot}]_i)$
$+ 3.4 \cdot [G_\alpha GTP\_AC] \cdot [ATP_{tot}]_i / (0.315 + [ATP_{tot}]_i) - 0.005 \cdot [PDE] \cdot [cAMP] / (0.0013 + [cAMP])$ S(77)

PKA activation by cAMP

The schematic presentation of reaction (a half of the dimer) is shown in the following figure, in which the half of the dimer PKA is shown. Cat_{123} represents the instantaneous equilibrium of sequential cAMP (A) bindings to the regulatory subunit (R), and the dissociation of the regulatory

unit from the catalytic subunit (C in this scheme) and Cat_{45} represents the binding of protein kinase inhibitor (pki) to C.

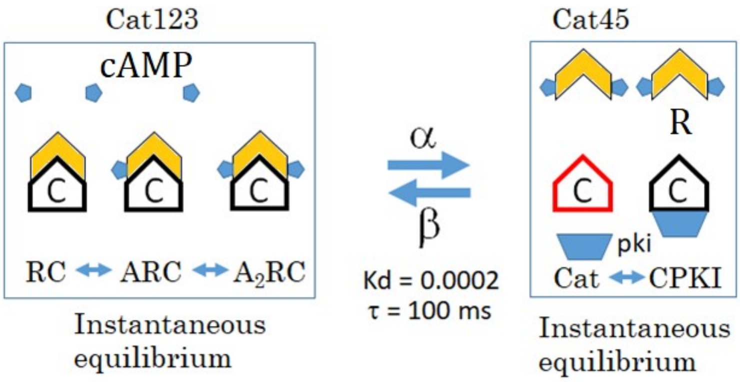

The mass conservation of the catalytic subunit of pKA is indicated by Eq. S(78), while Eq. S(79) indicates the composition of total cAMP in the Akinase system.

$[C_{tot}] = [PKA_{tot}] \cdot 2 = [RC] + [ARC] + [A_2RC] + [Cat] + [C\_PKI] \qquad [PKA_{tot}] = 0.001$ S(78)

$[cAMP_{tot}] = [cAMP] + ([ARC] + 2 \cdot [A_2RC] + 2 \cdot [A_2R])$ S(79)

In the original Saucerman model, the following instantaneous binding reactions are assumed.

$[RC] = Kd_{12} \cdot [ARC] / [cAMP] \qquad Kd_{12} = 0.008$ (mM) S(80)

$[ARC] = Kd_{12} \cdot [A_2RC] / [cAMP]$ S(81)

$[A_2RC] = [Cat] \cdot [A_2R] / Kd_3 \qquad Kd_3 = 0.009$ (mM) S(82)

$[C\_PKI] = [PKI_{tot}] \cdot [Cat] / (Kd_4 + [Cat]) \qquad Kd_4 = 0.0000002$ (mM) S(83)

In the present study, the instantaneous dissociation of A_2RC into C (Cat) and A_2R described in Eq. S(82) is calculated using forward and backward rates, α and β, where,

$$\beta = \alpha \cdot Kd_3 \qquad \alpha = 20 \quad (ms^{-1}) \qquad \text{S(84)}$$

New Lumped Model of PKA

<u>Catalytic subunit</u>

$$Cat_{123} \underset{\beta}{\overset{\alpha}{\rightleftarrows}} Cat_{45}$$

<u>Simplifications of the model</u>

In the present study, a time-dependent step is introduced between Cat_{123} and Cat_{45} The time-dependent step is only assumed on a technical reason; to simplify the computation to solve 6 first-degree simultaneous equations (2 mass conservation equations; Eqs. S(78) - (79) and 4 quasi-

equilibrium equations; Eqs. S(80) - S(83)) with 6 variables ([Cat], [RC], [ARC], [A_2RC], [A_2R] and [C_PKI]). Biophysically, the model is also simplified by neglecting additional state transitions, which might occur in reality. For example, cyclic AMP might dissociate from regulatory subunits within the conformation of Cat45.

Cat_{123}

From Eqs. S(81) - (83), the instantaneous equilibrium in Cat_{123} = RC + ARC + A_2RC is determined. Firstly, [A_2RC] fraction is determined by Eq. S(82).

$$[A_2RC] = Cat_{123} / ((Kd_{12} / [cAMP])^2 + (Kd_{12} / [cAMP]) + 1) \quad S(85)$$

Here, Kd_{12} is 0.008 (mM) and [cAMP] value at the previous time step is used in Eq. S(85) for the determination of [A_2RC]. (Only [$cAMP_{tot}$] but not [cAMP] is given by the numerical integration. [cAMP] needs to be calculated from Eq. S(79) using ARC A_2RC and ARC at the previous time step.)

Secondly, RC and ARC are determined by Eqs. S(81) and S(82) using [A_2RC] for calculation of Cat_{123} in Eq. S(88).

$$[RC] = (Kd_{12} / [cAMP]) \cdot [ARC] \quad S(86)$$

$$[ARC] = (Kd_{12} / [cAMP]) \cdot [A_2RC] \quad S(87)$$

$$[Cat_{123}] = [Cat_1 + Cat_2 + Cat_3] = [RC] + [ARC] + [A_2RC] \quad S(88)$$

$$[Cat_{123}] = (Kd_{12} / [cAMP])^2 [A_2RC] + Kd_{12} / [cAMP] \cdot [A_2RC] + [A_2RC] \quad S(89)$$

$$[Cat_{123}]= ((Kd_{12} / [cAMP])^2 + (Kd_{12} / [cAMP]) + 1) \cdot [A_2RC] \quad S(90)$$

Cat45 Inhibition of catalytic subunit by PKI

In the formulation of Kuzumoto model, the quasi-equilibrium is assumed using a dissociation constant Kd_4 = 0.000002.

$$Cat + PKI \leftrightarrow C\_PKI$$

$$[Cat_{45}] = [Cat_4 + Cat_5] = [Cat] + [C\_PKI] \quad S(91)$$

$$[C\_PKI] = [Cat_{45}] - [Cat] \quad S(92)$$

$$[C\_PKI] = [PKI_{tot}] \cdot [Cat] / (Kd_4 + [Cat]) \quad Kd_4 = 0.0000002 \quad (mM) \quad S(93)$$

From Eq. S(92) and S(93),

$$([Cat_{45}] - [Cat]) \cdot ([Cat] + Kd_4)$$
$$= [PKI_{tot}] \cdot [Cat] - [Cat]^2 + Kd_4 \cdot [Cat] + [Cat_{45}] \cdot [Cat] + Kd_4 \cdot [Cat_{45}] = [PKI_{tot}] \cdot [Cat]$$

$$[Cat]^2 - [Cat_{45}] \cdot [Cat] + [Cat] \cdot [PKI_{tot}] + [Cat] \cdot Kd_4 - [Cat_{45}] \cdot Kd_4 = 0$$

$[Cat]^2 + ([PKI_{tot}] - [Cat_{45}] + Kd_4) \cdot [Cat] - Kd_4 \cdot [Cat_{45}] = 0$ S(94)

Solution of the 2nd-degree equation

[Cat] = {-b + math.sqr(b^2 - 4ac)} / 2a

a = 1

$b = ([PKI_{tot}] - [Cat_{45}] + Kd_4)$ S(95)

$c = - Kd_4 \cdot [Cat_{45}]$

The time-dependent state transition between Cat_{123} ←→ Cat_{45}

The model is now simplified into a lumped two state model.

$$Cat_{123} \underset{\alpha'}{\overset{\beta'}{\rightleftarrows}} Cat_{45}$$

$\tau = 1 / (\alpha' + \beta')$ S(96)

Using rate constants, α and β, assumed for the reactions between Cat_3 and Cat_4, the forward and backward fluxes between Cat_{123} and Cat_{45} are calculated as follows;

Forward Flux = $\beta \cdot [A_2RC]$ S(97)

From Eq. S(84)

$\beta \cdot [A_2RC] = \beta \cdot Cat_{123} / ((Kd_{12} / [cAMP])^2 + (Kd_{12} / [cAMP]) + 1)$

Forward rate constant $\beta' = \{\beta / ((Kd_{12} / [cAMP])^2 + (Kd_{12} / [cAMP]) + 1)\}$ (ms^{-1}) (34) S(98)

Backward Flux = $\alpha \cdot [Cat][A_2R]$ S(99)

Since $[A_2R] = [Cat_{45}]$,

Backward rate constant $\alpha' = \alpha \cdot [Cat]$ (ms^{-1}) S(100)

From Eq. S(84), β can be determined when α is provided, and subsequently, α' and β' are determined.

Initial values for the Euler integration

Parameter	Value	Parameter	Value
Vm	-66.447116401342214	I1NCX_blk	0.171664945867262
TnChCa	0.10447717129344221	I2NCX_blk	0.68159262452779745
CaMCa	0.0003075838194616453	c1	0.28621564970090407
bufferSRCa	0.0022493020872266464	c2	0.6618729884176402

Catot_jnc	0.27887964039384816	pr	0.0074631215577577181
Catot_iz	0.11543731073765753	s	0.95571772650734277
Catot_blk	0.10713873825176626	s_slow	0.95571772657751919
Nai	5.7835890034878989	O_TM	0.000025812378966131479
Ki	117.55390194536011	I2_TM	0.039145817711564689
Ca_SRup	0.903023147511798	Is_TM	0.64483536379864259
Catot_SRrl	2.3938173987628852	a_Kur	0.0033102437586842066
Yooo	0.000040613205495200059	i_Kur	0.97651353426156917
Yooc	0.00004098083266229187	TSCa3	0.0044381619418831396
Ycoo	0.000093395229562045015	TSCa3W	0.0001130611639904043
Ycoc	0.99978802625560148	TSCa3S	0.000050253626145520288
Ycco	0.0000000083592508382794764	TSS	0.000134045303367519
Yoco	0.0000000017708845117738384	TSW	0.000069880364775602439
Yocc	0.0000000071297350978473211	crsBrLnXw	0.9699
Yco_iz	0.99989743973372536	crsBrLnXp	0.96459
Yoc_iz	0.0000000054033757677464432	R_ip3	0.46617993859032436
Yoo_iz	0.0000815975354013338	O_ip3	0.0081974458936357464
Yco_blk	0.99991079283327222	I_1_ip3	0.16267555056316188
Yoc_blk	0.0000000043136867481938156	I_2_ip3	0.22732673455466326
Yoo_blk	0.00008159862509035334	A_ip3	0.13559032296878337
y1_IKr	0.0011330794896614053	IP3_m	0.015
y2_IKr	0.0011332895929810937	hIP3R_t	0.99999999999167333
y3_IKr	0.98562965758986421	ARS464	0.0000000000054552197286606261
Pbspm	0.88798165746058544	ARS301	0.00000033958594680314912
E1NCX_jnc	0.44878015194210857	GsaGTPtot	0.000021287282946520791
I1NCX_jnc	0.11630535096826131	Gsbg	0.000022090314190328611
I2NCX_jnc	0.43086843058119384	GsaGDP	0.00000080303984373160733
E1NCX_iz	0.32718383921221383	cAMPtot	0.00014833521997096224
I1NCX_iz	0.13787993637331128	Cat45	0.00008089376821908432
I2NCX_iz	0.53284422579114621	y_PKA	0.11693851726524969
E1NCX_blk	0.14617284203483963	hIP3R_t	0.99999999999167333

References (reference numbers in supporting materials correspond to those in the main text)

Himeno, Y.; Asakura, K.; Cha, C. Y.; Memida, H.; Powell, T.; Amano, A.; Noma, A., A human ventricular myocyte model with a refined representation of excitation-contraction coupling. *Biophys J* **2015,** 109, (2), 415-27.

Hinch, R., A mathematical analysis of the generation and termination of calcium sparks. *Biophys J* **2004,** 86, (3), 1293-307.

Hinch, R.; Greenstein, J. L.; Tanskanen, A. J.; Xu, L.; Winslow, R. L., A simplified local control model of calcium-induced calcium release in cardiac ventricular myocytes. *Biophys J* **2004,** 87, (6), 3723-36.

Asakura, K.; Cha, C. Y.; Yamaoka, H.; Horikawa, Y.; Memida, H.; Powell, T.; Amano, A.; Noma, A., EAD and DAD mechanisms analyzed by developing a new human ventricular cell model. *Prog Biophys Mol Biol* **2014,** 116, (1), 11-24.

Yan, D. H.; Ishihara, K., Two Kir2.1 channel populations with different sensitivities to Mg(2+) and polyamine block: a model for the cardiac strong inward rectifier K(+) channel. *J Physiol* **2005,** 563, (Pt 3), 725-44.

Pandit, S. V.; Clark, R. B.; Giles, W. R.; Demir, S. S., A mathematical model of action potential heterogeneity in adult rat left ventricular myocytes. *Biophys J* **2001,** 81, (6), 3029-51.

Bondarenko, V. E.; Szigeti, G. P.; Bett, G. C.; Kim, S. J.; Rasmusson, R. L., Computer model of action potential of mouse ventricular myocytes. *Am J Physiol Heart Circ Physiol* **2004,** 287, (3), H1378-403.

Sneyd, J.; Dufour, J. F., A dynamic model of the type-2 inositol trisphosphate receptor. *Proc Natl Acad Sci U S A* **2002,** 99, (4), 2398-403.

Saucerman, J. J.; Brunton, L. L.; Michailova, A. P.; McCulloch, A. D., Modeling beta-adrenergic control of cardiac myocyte contractility in silico. *J Biol Chem* **2003,** 278, (48), 47997-8003.

Kuzumoto, M.; Takeuchi, A.; Nakai, H.; Oka, C.; Noma, A.; Matsuoka, S., Simulation analysis of intracellular Na+ and Cl- homeostasis during beta 1-adrenergic stimulation of cardiac myocyte. *Prog Biophys Mol Biol* **2008,** 96, (1-3), 171-86.

Miyakawa, T.; Maeda, A.; Yamazawa, T.; Hirose, K.; Kurosaki, T.; Iino, M., Encoding of Ca2+ signals by differential expression of IP3 receptor subtypes. *EMBO J* **1999,** 18, (5), 1303-8.

Okamoto, Y.; Takano, M.; Ohba, T.; Ono, K., Arrhythmogenic coupling between the Na+ -Ca2+ exchanger and inositol 1,4,5-triphosphate receptor in rat pulmonary vein cardiomyocytes. *J Mol Cell Cardiol* **2012,** 52, (5), 988-97.

Cooling, M.; Hunter, P.; Crampin, E. J., Modeling hypertrophic IP3 transients in the cardiac myocyte. *Biophys J* **2007,** 93, (10), 3421-33.

Doisne, N.; Maupoil, V.; Cosnay, P.; Findlay, I., Catecholaminergic automatic activity in the rat pulmonary vein: electrophysiological differences between cardiac muscle in the left atrium and pulmonary vein. *Am J Physiol Heart Circ Physiol* **2009,** 297, (1), H102-8.
